# Designing tailored maintenance strategies for systematic reviews and clinical practice guidelines using the Portfolio Maintenance by Test-Treatment (POMBYTT) framework

**DOI:** 10.1186/s12874-024-02155-z

**Published:** 2024-02-02

**Authors:** Michiel S. Oerbekke, Roy G. Elbers, Maarten J. van der Laan, Lotty Hooft

**Affiliations:** 1 Cochrane Netherlands, Julius Center for Health Sciences and Primary Care, University Medical Center Utrecht, Utrecht University, Utrecht, The Netherlands; 2 Knowledge Institute of the Dutch Association of Medical Specialists, Utrecht, The Netherlands; 3 Department of General Practice, Intellectual Disability Medicine, Erasmus MC, University Medical Center Rotterdam, Rotterdam, The Netherlands; 4 Department of Surgery, University Medical Center Groningen, Groningen, The Netherlands; 5 Julius Center for Health Sciences and Primary Care, University Medical Center Utrecht, Utrecht University, Utrecht, The Netherlands

**Keywords:** Clinical practice guidelines as topic, Systematic reviews as topic, Concept formation, Theoretical model, Need for updating, Maintenance

## Abstract

**Background:**

Organizations face diverse contexts and requirements when updating and maintaining their portfolio, or pool, of systematic reviews or clinical practice guidelines they need to manage. We aimed to develop a comprehensive, theoretical framework that might enable the design and tailoring of maintenance strategies for portfolios containing systematic reviews and guidelines.

**Methods:**

We employed a conceptual approach combined with a literature review. Components of the diagnostic test-treatment pathway used in clinical healthcare were transferred to develop a framework specifically for systematic review and guideline portfolio maintenance strategies.

**Results:**

We developed the Portfolio Maintenance by Test-Treatment (POMBYTT) framework comprising diagnosis, staging, management, and monitoring components. To illustrate the framework’s components and their elements, we provided examples from both a clinical healthcare test-treatment pathway and a clinical practice guideline maintenance scenario. Additionally, our literature review provided possible examples for the elements in the framework, such as detection variables, detection tests, and detection thresholds. We furthermore provide three example strategies using the framework, of which one was based on living recommendations strategies.

**Conclusions:**

The developed framework might support the design of maintenance strategies that could contain multiple options besides updating to manage a portfolio (e.g. withdrawing and archiving), even in the absence of the target condition. By making different choices for variables, tests, test protocols, indications, management options, and monitoring, organizations might tailor their maintenance strategy to suit specific contexts and needs. The framework’s elements could potentially aid in the design by being explicit about the operational aspects of maintenance strategies. This might also be helpful for end-users and other stakeholders of systematic reviews and clinical practice guidelines.

**Supplementary Information:**

The online version contains supplementary material available at 10.1186/s12874-024-02155-z.

## Background

Fifteen percent of the systematic reviews (SRs) [1] and eight percent of the recommendations in clinical practice guidelines (CPGs) [2] may be out of date within the first year after their publication. Over time, there could be changes in the evidence on the harms, benefits, and availability of interventions, and changes in important outcomes for instance [3]. Neglecting such changes could cause SR conclusions and CPG recommendations to become invalid, potentially leaving clinical practice sub-optimal. Updating thus seems a reasonable option to manage outdated SRs and CPGs. The problem of when and how to update SRs was highlighted more than one decade ago [4] and more than two decades ago for CPGs [3]. The Cochrane Collaboration provides guidance on when and how to update an SR [5, 6]. Furthermore, specific strategies to detect the need for updating were being developed for SRs, such as the Ottawa [7] and RAND methods [8], and for CPGs [3, 9–12]. Previous published systematic reviews provided overviews of such methods for both SRs [4] and CPGs [9, 13].

A large variety of strategies to assess when to update SRs or CPGs can be observed in the literature [13, 14]. Even within similar assessments, such as literature searches to identify new evidence, there is a variety in how the assessment is performed. For example, search strategies can be limited to specific journals [7, 8, 10] and publication type [7, 10]. The full search strategy of the original reviews can be updated [15], additional searches can be performed in a guideline database [10], experts can be consulted [3, 12], or studies can be tracked in trial registries [15, 16]. New strategies and insights about updating strategies are still being introduced, such as the concept of living SRs [17] and living CPG recommendations [18]. Current strategies may not be useful for the context, capabilities, or the needs of all organizations performing updates. Different choices can be made in designing strategies to accommodate for the different contexts, capabilities, and needs. For example, strategies with an extensive literature search for each key question could be too resource intensive for CPG developing organizations managing a large portfolio (i.e. a pool of SRs or CPGs that is managed by the organization). Such considerations might prevent adoption or cause revisitation of existing strategies and could partially explain why new strategies are still being reported. Cochrane, for example, has changed their updating principles on several occasions reflecting their experience that they were not yet able to constantly keep their entire portfolio of SRs up-to-date over time [15]. Furthermore, updating might not be the only option available to manage an outdated SR or CPG. Withdrawal or archiving could be suitable alternative options to maintain the portfolio of SRs or CPGs as well, where withdrawal completely removes the SR or CPG from the portfolio and archiving still allows end-users to access the information while no longer actively maintained. It seems, rather, that there could be a need for guidance to design and tailor maintenance strategies instead of updating strategies.

A framework with explicit underlying key components and elements for designing portfolio maintenance strategies appears to be missing at present. A new framework therefore should identify and explain these key components and elements in the context of a maintenance strategy, potentially enabling organizations to tailor a strategy according to their context, capabilities, needs and available resources. We aimed to develop and describe such a theoretical framework for designing and tailoring maintenance strategies for managing portfolios of SRs and CPGs.

## Methods

A literature review was conducted to gain a comprehensive overview of considerations, signals, or indicators for updating SRs and CPGs. The literature review (methodology reported in Additional file 1) is not exhaustive, as we did not need to capture all data on every domain. During the data-extraction we observed that other management options were available besides (not) updating. For example, withdrawing an SR or CPG. While exploring the extracted data thereafter, we observed a supposed interrelatedness between some considerations, signals, and indicators. Through discussion among the authors, we believed that the interrelatedness and the availability of multiple management options had an analogy to a diagnostic test-treatment pathway in the clinical care setting. In a test-treatment pathway, medical tests are linked to management actions through pathways so that test results guide clinical management [19]. We envisioned a parallel scenario where considerations, signals, and indicators guide the selection of appropriate management actions for SR and CPG maintenance. We therefore transferred the diagnosis, staging, management, and monitoring concepts of a diagnostic test-treatment pathway to develop a theoretical framework for designing and tailoring SR and CPG maintenance strategies. We recognize that alternative conceptual frameworks or constructs could have been considered as well, however the analogy to a diagnostic test-treatment pathway resonated with us due to its apparent suitability to represent how considerations, signals, and indicators could be linked to management. The extracted data from our literature review were qualitatively analyzed and these results were used to provide some possible examples of key elements in the framework. Thus, data from the literature review both directed us to use a diagnostic test-treatment strategy analogy and provided examples for the framework’s elements. To explicitly clarify the components and elements in the framework, we describe both a clinical healthcare example and a CPG maintenance scenario. The 2018 European Society of Cardiology and European Society of Hypertension guideline for the management of arterial hypertension was used as clinical example [20]. The CPG maintenance strategy scenario was based on considerations and signals found in the literature review, however, modified for illustrative purposes. Tables concerning the clinical example and the CPG maintenance scenario represent subsequent steps in the diagnostic test-treatment pathway. Results from our literature review were mapped at our own discretion to the specific test-treatment components of the maintenance strategy to provide examples, even though the extracted data may have been described for other purposes in the original references.

## Results

### Literature search

Fifty-four references were included. The study selection flow (Figure A1 in Additional file 1) and reasons for exclusion of full-text references are reported in Additional file 1 (Table A1). General characteristics of the included studies are described in Additional file 1 (Table A2). Results from the literature review are provided as possible examples for elements in the framework in Additional File 1 (Tables A3 to A9).

**Fig. 1:**
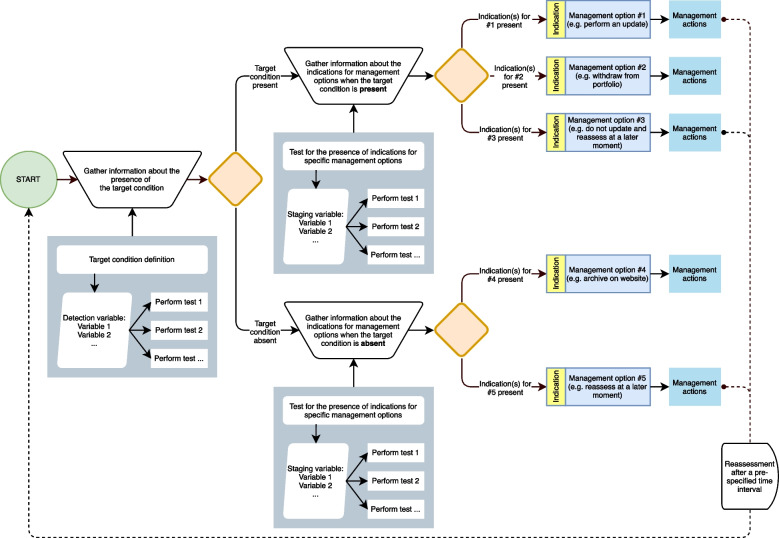
The Portfolio Maintenance by Test-Treatment framework. The figure shows the framework depicted as a flow diagram in analogy to a diagnostic test-treatment pathway. Tests are performed (grey boxes, not outlined), choices are made (outlined orange diamonds), management options (outlined blue boxes) are selected based on indications (outlined yellow boxes), subsequent management actions are performed (blue boxes, not outlined), and predefined time intervals are used for reassessments (dashed line)

**Table 1:** An overview of test-treatment pathway components compared to an SR or CPG portfolio maintenance strategy

Component	Diagnostic test-treatment pathway	SR or CPG portfolio maintenance strategy
***Diagnosis*** *(see “*Diagnosis*” section)*	A “prevalent disease or condition” is diagnosed. Diagnosis requires a clear definition of the target condition (i.e. the disease or condition) and the use of tests to assess the likelihood of its presence or absence. In a clinical scenario, diagnostic tests include anamnesis (medical history) along with other clinical tests, such as physical examination or imaging. By setting a threshold within the test, healthcare professionals can determine the likelihood of the disease being present	The diagnosis of “prevalent outdatedness” involves the use of tests to determine the target condition’s likelihood of presence or absence. Similar to a clinical test-treatment pathway, these tests help to determine whether the target condition is likely to be present or absent. It is important to define outdatedness in advance. A threshold within a test can then be established to assess whether outdatedness is likely to be present or absent
***Staging*** *(see “*Staging*” section)*	Staging of a disease or condition is conducted to evaluate its stage or degree of severity. Information necessary for staging is gained with tests. Within these tests, thresholds are used to indicate the severity of the disease or condition	When “prevalent outdatedness” is diagnosed, its severity is assessed using tests. Several stages or degrees of outdatedness might result in different decisions regarding appropriate management options. Thresholds within the tests serve as criteria for categorizing stages or severity of outdatedness. Staging can also be performed when the systematic review (conclusion) or clinical practice guideline (recommendation) is still op to date
**Management** *(see “*Management*” section)*	There are specific indications to select management options. Staging provides valuable information to determine which management option is most appropriate. Once a management option is chosen, actions are performed to initiate and carry out the appropriate care	Management options, such as updating, not updating, or withdrawing the SR or CPG have specific indications. Staging provided information to decide which management option is appropriate. Additionally, organizations may have several other management options (e.g. archive, re-endorse). Once the management option is selected, actions are performed specific for that option. For example, an update of the SR or CPG is initiated and carried out
**Monitoring** *(see “*Monitoring*” section)*	Patients are periodically seen during follow-up visits as part of their ongoing care. The purpose of these visits is to monitor the disease or condition over time and evaluate the success of the chosen management option, detect signs of disease recurrence, or identify any disease progression	Periodic evaluation of SRs or CPGs can be performed to assess whether there is an occurrence, recurrence, or progression of outdatedness. This periodic evaluation is especially relevant when the initial staging of the target condition did not meet the threshold for selecting a specific management option (e.g. updating)

*CPG* Clinical Practice Guideline

*SR* Systematic review

**Table 2:** Glossary of terms used in the conceptual maintenance strategy for systematic reviews (SRs) and clinical practice guidelines (CPGs)

Component	Element	Description as used in the maintenance of SRs and CPGs
**Diagnosis**	*Target condition*	The predefined condition of the SR (conclusion) or CPG (recommendation) that is to be detected by one or multiple tests
	*Detection variable*	A variable or characteristic of the predefined target condition on which a detection test specifically measures information
	*Detection test*	A test to measure or obtain information on a detection variable to determine whether the target condition is likely to be present or absent
	*Detection test protocol*	The protocol or manner how the detection test is carried out to measure the detection variable or obtain information
	*Detection test threshold*	A predefined threshold for the detection variable in a detection test to define the presence or absence of the target condition
**Staging**	*Staging variable*	A variable or characteristic of the stage or severity of the target condition on which a staging test specifically measures information. A staging variable can also concern a status or circumstance not related to the target condition itself
	*Staging test*	A test to measure or obtain information on a staging variable to determine the stage or severity of the target condition. A staging test may also capture a status or circumstance not related to the target condition itself
	*Staging test protocol*	The protocol or manner how the detection test is carried out to measure the staging variable or obtain information
	*Staging test threshold*	One or more predefined thresholds for the staging variable in a staging test to define the stage, severity, status, or circumstance
**Management**	*Management indication*	A specific stage, severity, status, or circumstance that guides the decision to the appropriate management option
	*Management options*	A predefined approach to handle an SR (conclusion) or CPG (recommendation) when the target condition is present or absent, and depending on the specific stage, severity, status, or circumstance. The choice for a management option is guided by its indication(s)
	*Management actions*	Actions that follow from the decision for a specific management option. The actions performed to carry out the chosen management of the SR (conclusion) or CPG (recommendation)
**Monitoring**	*Recurrence*	The recurrence of the predefined target condition after an update. Monitoring is used to detect recurrences
	*Progression*	The progression of the stage, status, or severity of the predefined target condition. Monitoring is used to detect progression until a threshold for a specific management option is reached (e.g. update)

*CPG* Clinical Practice Guideline

*SR* Systematic review

### A theoretical framework for portfolio maintenance strategies

The Portfolio Maintenance by Test-Treatment (POMBYTT) framework is shown in Fig. 1. The theoretical POMBYTT framework is intended to help design and tailor maintenance strategies for portfolios consisting of SRs or CPGs. Components of a diagnostic test-treatment pathway are transferred to a portfolio maintenance context: diagnosis, staging, management, and monitoring (Table 1). These concepts in the framework are outlined in Additional file 1 (Figure A2). Specific terminology is used throughout the description of the framework and a glossary of terms can be found in Table 2.

#### Diagnosis

The target condition must be defined before it can be detected with diagnostic tests. Let’s consider the example of determining whether a CPG recommendation is outdated. In this care we can define a recommendation as outdated when at least one new relevant peer-reviewed article is published after the previous search date. It is important to have a specific definition that outlines the unit of analysis. In the context of SR or CPG maintenance strategies, the unit of analysis can be the entire SR or CPG, or it can focus on the SR conclusion or CPG recommendation. Like diagnosing a medical condition in clinical practice, we need one or more detection variables (Table A3 in Additional file 1) that provide information about the presence or absence of the target condition. In the provided example in Table 3, the detection variable was “*new available evidence*” but it is worth noting that other detection variables can be used depending on the specific context. To measure these detection variables, we can use detection tests (Table A4 in Additional file 1). For example, a literature search in a database like MEDLINE can be used as a test to measure the detection variable “*new available evidence*”. The test protocol for the literature search can vary, including the choice of using multiple databases, limiting the search to specific databases, or even limiting to a few specific journals. Additionally, literature selections can be performed by a single person or in a double-blind fashion and any selection procedure in between.

**Table 3:** Example of the target condition and diagnostic test in a clinical example and CPG scenario. The CPG scenario is based on considerations and signals found in our literature search, however, modified for illustrative purposes

	**Clinical healthcare example**	**GPG maintenance scenario**
*Objective*	To detect whether there is arterial hypertension	To detect whether a recommendation is outdated
*Target condition definition*	We consider high blood pressure to be prevalent when there is at least 140 mmHg systolic arterial pressure over 90 mmHg diastolic arterial pressure	We consider the recommendation to be outdated when at least one new relevant peer-reviewed article is published after the previous search date
*Detection variable*	Pressure on artery walls	New available evidence
*Detection test*	Auscultatory sphygmomanometer	Literature search in MEDLINE
*Detection test protocol*	Three blood pressure measurements are taken one to two minutes apart after the patient is seated in a quiet environment for five minutes. The last two blood pressure measurements are averaged	A sensitive search string for MEDLINE is constructed. Search results are screened on title and abstracts by two independent assessors. Conflicts are resolved by a third independent assessor. The resulting potentially relevant articles are read and selected by two assessors independently based on the full text. A third assessor resolves any conflict in the full text selection
*Detection test threshold*	140 mmHg systolic arterial pressure over 90 mmHg diastolic arterial pressure	Any new peer-reviewed scientific article published after the previous search date

**Table 4:** Example of a single staging test and its thresholds in the presence of the target condition. The CPG scenario is based on considerations and signals found in our literature search, however, modified for illustrative purposes

	**Clinical healthcare example**	**GPG maintenance scenario**
*Target condition*	Arterial hypertension is present (≥ 140/90 mmHg)	The recommendation is outdated (There was new peer-reviewed scientific evidence published)
*Objective*	To detect the severity, stage, or status of the prevalent hypertension so that an appropriate management option can be selected	To detect the severity, stage, or status of the prevalent outdatedness so that an appropriate management option can be selected
*Staging variable*	Magnitude of pressure on artery walls in a free-living setting	Likelihood of potential changes in the strength of the recommendation
*Staging test*	Ambulatory blood pressure monitoring	Survey among experts
*Staging test protocol*	The patient receives a blood pressure measuring device to wear over the course of 24 h. The device is programmed to record the blood pressure once every 30 min. Blood pressure measurements are averaged for daytime and nighttime	A survey among experts is performed by using the results from the literature selection. Experts are given the current recommendation and literature, while being asked whether the newly identified literature is likely to change the recommendation’s strength requiring a dichotomous answer (yes/no)
*Staging test thresholds*	**Grade 1:** 140–159 mmHg systolic and/or 90–99 mmHg diastolic arterial pressure **Grade 2:** 160–179 mmHg systolic and/or 100–109 mmHg diastolic arterial pressure **Grade 3:** ≥ 180 mmHg systolic and/or ≥ 110 mmHg diastolic arterial pressure	**Very unlikely:** 0–20% of the surveyed experts indicated that the new evidence is likely to change the strength of the recommendation when updated **Reasonably unlikely:** 20–40% of the surveyed experts indicated that the new evidence is likely to change the strength of the recommendation when updated **Unclear:** 40–60% of the surveyed experts indicated that the new evidence is likely to change the strength of the recommendation when updated **Reasonable likely:** 60–80% of the surveyed experts indicated that the new evidence is likely to change the strength of the recommendation when updated **Very likely:** 80–100% of the surveyed experts indicated that the new evidence is likely to change the strength of the recommendation when updated

A detection test threshold (Table A5 in Additional file 1) is used to determine whether it is likely that the target condition is present or not. The threshold determines how the target condition is defined. In Table 3, the threshold to detect the target condition was any new relevant peer-reviewed article (i.e. ≥ 1). If the threshold was increased to at least 3 new relevant peer-reviewed articles a different definition of the target condition is detected (i.e. outdated when ≥ 3 new relevant articles). See Table 3 for a clinical example and a CPG scenario.

**Table 5:** Example of staging and management when the target condition is absent

		**Clinical healthcare example**	**GPG maintenance scenario**
***Target condition***		Arterial hypertension is **absent** (< 140/90 mmHg)	The recommendation is **not outdated** (There was no new peer-reviewed scientific evidence published)
***Staging***	*Staging variable*	Magnitude of pressure on artery walls in a free-living setting	Practice variation
	*Staging test*	Ambulatory blood pressure monitoring	Data registry analysis
	*Staging test protocol*	The patient receives a blood pressure measuring device to wear over the course of 24 h. The device is programmed to record the blood pressure each 30 min. Blood pressure measurements are averaged for daytime and nighttime	Data from registries are obtained. Variables concerning the CPG recommendation are analyzed in statistical software to show whether there are deviations from the recommendation in clinical practice
	*Staging test thresholds*	**Optimal:** < 120 mmHg systolic and/or < 80 mmHg diastolic arterial pressure **Normal:** 120–129 mmHg systolic and/or 80–84 mmHg diastolic arterial pressure **High normal:** 130–139 mmHg systolic and/or 85–89 mmHg diastolic arterial pressure	**Neglectable practice variation:** No or some deviations from the recommendation are observed, however it was judged that these deviations are not of importance or that the observed deviations are not necessarily unwanted **Considerable unwanted practice variation:** It was judged that most of the observed deviations from the recommendation are unwanted
***Management***	*Option #1, when: indications*	**Do not provide an intervention, when:** *Optimal OR normal blood pressure is present*	**Re-assess at a later point in time, when:** *Neglectable practice variation is present*
	*Option #2, when: indications*	**Advice on lifestyle changes, when:** *High normal blood pressure is present*	**Archive, when:** *Neglectable practice variation is present AND the clinical field signals that guidance is no longer needed*^a^
	*Option #3, when: indications*	**Advice on lifestyle changes and consider drug prescription, when:** *High normal blood pressure is present* AND *very high cardiovascular risk profile especially with coronary artery disease is present*^a^	**Update, when:** *Considerable unwanted practice variation is present*

^a^Different staging variables and tests are required to provide enough information for the several indications to choose an appropriate management option

#### Staging

The staging process occurs after determining whether the target condition is present or absent (Figure A2 in Additional file 1). The goal is to gain information about the severity, status, or stage. This is done by utilizing one or multiple staging variables (Table A6 in Additional file 1), staging tests (Table A7 in Additional file 1), and staging thresholds (Table A8 in Additional file 1).

**Table 6:** Examples of management options and their indications in the presence of the target condition

	**Clinical healthcare example**	**CPG maintenance scenario**
*Target condition*	Arterial hypertension is present (≥ 140/90 mmHg)	The recommendation is outdated (There was new peer-reviewed scientific evidence published)
*Objective*	To choose an appropriate management option in the presence of the target condition using the information gathered from staging	To choose an appropriate management option in the presence of the target condition using the information gathered from staging
*Management option #1, when: indications*	**Advice on lifestyle changes, when:** *Grade 1 blood pressure* AND *low to moderate cardiovascular risk profile without cardiovascular disease, renal disease, or hypertension-mediated organ damage*^a^	**Update the recommendation, when:** *New evidence is very likely cause changes in the strength of the recommendation* OR *new evidence is very likely cause changes in the direction of the recommendation* OR *new evidence indicates a new recommendation should be added* OR *new evidence indicates an existing recommendation should be removed*^a^
*Management option #2, when: indications*	**Advice on lifestyle changes and drug prescription after 3–6 months, when:** *Grade 1 blood pressure* AND *low to moderate cardiovascular risk profile without cardiovascular disease, renal disease, or hypertension-mediated organ damage* AND *blood pressure was not controlled after 3–6 months of lifestyle interventions*^a^	**Do not update the recommendation, but reassess at a later point in time, when:** *The new evidence is very unlikely or somewhat unlikely to cause changes in the strength or direction of the recommendation* OR *no new evidence was found* OR *a valid justification is provided to postpone an update*^a^
*Management option #3, when: indications*	**Advice on lifestyle changes and immediate drug prescription, when:** *(Grade 1 blood pressure* AND *high to very high cardiovascular risk profile with cardiovascular disease, renal disease, or hypertension-mediated organ damage)*^a^ OR *grade 2 blood pressure* OR* grade 3 blood pressure*	**Withdraw the recommendation, when:** *The clinical field signals that guidance is no longer needed* OR *new evidence indicates that an intervention should be de-implemented*^a^* OR registry data shows that the recommendations were fully implemented*

^a^Different staging variables and tests are required to provide enough information for the several indications to choose an appropriate management option. A sphygmomanometer or ambulatory blood pressure monitor only provides information about the level of blood pressure. Similarly, a survey for the CPG recommendation that was solely intended to assess possible changes in the strength and direction does not provide information about de-implementation or whether new recommendations should be added

Staging tests are used to measure information on the staging variable. Staging thresholds are defined in order to define the different stages or severity. The information obtained from the staging tests, along with the staging thresholds, guide the decision-making process towards an appropriate management option. Identical to detection tests, staging tests have variations in the test protocol and changing the thresholds also changes the definition of the stage, status, or severity. Table 4 provides a clinical example and a CPG scenario offering an understanding of the staging process.

It can still be important to perform staging tests when the target condition is absent, as several management options might still be available (see Table 5). A specific status or circumstance may be present that guides the management decision towards a specific management option.

#### Management

A management option is chosen once the severity, stage, or status is reasonably determined. Multiple management options can be available besides just updating an outdated SR or CPG. Such options can include withdrawal, archiving, choosing not to update, or deferring an update to a later time. Similarly, when the target condition is not present, there can be multiple management options available as well (Table 5).

For example, if certain indicators are met, such as the CPG recommendation being fully implemented and there is minimal practice variation, it may be appropriate to archive the SR or CPG. Each management option has its own specific indications (Table A9 and Figure A2 in Additional file 1). The presence or absence of these indications, as evaluated using staging tests, guide the decision for specific management options. This process is similar to selecting appropriate management in clinical practice (see Table 6).

Once a management option is chosen, subsequent actions are undertaken to carry out the management option. These actions can be described in detail and can usually be found in guideline development methodology handbooks (e.g. updating procedures). Available management options can have a unique set of subsequent actions. For instance, archiving a CPG requires different actions compared to withdrawing or (not) updating a CPG. Additionally, it’s worth considering that the set of management actions may differ between organizations for the same management option (e.g. updating).

#### Monitoring

In clinical practice, patients are usually followed over time to assess whether the selected management succeeded, to identify disease recurrence, or to assess disease progression. Similarly, SRs or CPGs in the portfolio can be monitored through cyclical assessments (see Figure A2 in Additional file 1). The cyclical assessments start by pre-specifying a time interval on which these reassessments take place. This means that the expiration of the prespecified time interval triggers a new cycle of assessments in the maintenance strategy rather than indicating that the SRs or CPGs are outdated. The choice of appropriate time intervals is essential. Prespecified time intervals should be long enough to allow for the development of new cases, recurrences, or progression, but not so long to cause excessive harm when the target condition had already developed early in the interval. If time intervals are too short, frequent assessments are resource intensive relative to the benefits. Too long intervals might lead to harmful consequences due to delayed identification of evolving conditions or outdated conclusions and recommendations.

### Designing and tailoring a maintenance strategy

Maintenance strategies within organizations can potentially be designed and tailored according to the needs and capabilities of the organization by using the concepts of a test-treatment pathway. Table A10 in Additional file 1 provides a blank process description table to design or tailor a maintenance strategy. Some detection and staging variables could provide more predictive information than others. The measurement of information on those variables may require more resources due to the nature of the tests or test protocols involved. If the organization is not capable or willing to spend such resources (e.g. budget, work force, time), a less resource intensive variable, test, or test protocol may be selected to obtain the information. However, this trade-off might result in a reduced predictive strength for the presence or absence of the target condition and management indications. Three examples of tailored maintenance strategies are provided in Additional file 1 (Tables A11-13 and Figures A3-5, respectively).

In these hypothetical scenarios, different choices were made between strategies leading to variations in how the target condition was defined, the selection of different detection and staging variables and tests, differences in management indications, and the availability of different management options. These variations resulted in different process flows, even though the underlying concepts and elements within the framework remain the same.

## Discussion

### The framework in context

Initially, we observed a large variety of updating strategies being reported in the literature [3, 9–12, 15, 21–24]. These strategies may not directly be applicable or adopted by other organizations, as organizations probably must consider various factors related to their context, capabilities, needs, and available resources when designing or tailoring their maintenance strategy. Different choices for those considerations may result in different strategies being implemented. The POMBYTT framework introduces key components in maintenance strategies based on a diagnostic test-treatment pathway. It provides theoretical guidance to designers, emphasizing the explicit consideration of key elements in the framework and thus operational aspects in the strategy. First, it prompts consideration about how the target condition (e.g. outdatedness) is defined, ensuring clarity in its definition. Next, it guides the determination of how the presence or absence of the target condition is assessed, including establishing the threshold for decision-making. Furthermore, the framework guides considerations for selecting appropriate management options based on indications, how to test for these indications and establishing staging thresholds. Additionally, it guides considerations about how monitoring processes can be performed. The components and elements may also be useful for stakeholders and end-users of SRs and CPGs. For instance, understanding the diagnostic and staging components can be helpful for clinicians and local protocol developers to informally screen the CPGs and SRs they consult. This might eventually result in stronger signals from the clinical field to organizations maintaining SRs and CPGs, indicating whether an SR or CPG is considered outdated for practice.

Some of the reported strategies lead to multiple management options [9, 10, 15, 16, 25]. Most of these options seem to focus on variations of (not) updating. For example, *“don’t update”*, “*don’t update yet*”, “*to be updated*”, or “*update now”* [16], and *“prepare update”*, *“update pending”*, *“no update planned”,* or *“up to date”* [15]. Other strategies lead to *“exclude”*, *“no update”*, *“exceptional update”*, and *“start regular update”* [9], or *“don’t update”*, “*don’t update yet*”, and “*to be updated*” [16]. This may reflect the different needs or preferences for management options within organizations. Through the POMBYTT framework it becomes prevalent that there might be more management options available in the strategy than (not) updating, even when the target condition is absent. For example, re-endorsing, archiving, or withdrawing. The theoretical framework reveals that the question ‘*when to update?*’ is only one part of a maintenance strategy, which leads to the updating management option. The question ‘*how to manage?*’ is probably a more encompassing question in the context of portfolio maintenance. Furthermore, the framework could potentially aid in adapting existing strategies to the needs and capabilities of an organization. The existing strategy could be mapped to the framework (e.g. by using the Table A10 in Additional file 1) and changes or additions to the strategy can be made in line with the organization’s context, needs, capabilities, and/or resources.

It can be argued that the living SR or CPG is a competing or complementary concept to the POMBYTT framework. However, it is possible to map the elements of living SRs or CPGs to the theoretical POMBYTT framework. In the case of a living CPG recommendation, updates are made when new relevant evidence becomes available [18]. Based on this, we can deduce that the definition of the target condition could be *‘outdatedness of a recommendation is present when there is new relevant evidence’*, the detection variable could be *‘new evidence’*, the detection test could be a *‘literature search and selection’*, and the detection threshold is *‘any new relevant evidence’*. Further guidance suggests a possible staging test where the CPG panel discusses the potential effect of changes in the body of evidence on the recommendation [26]. This approach is also seen in other living CPG literature, where an expert panel could be considered as a staging test using ‘*the content of the recommendation changes* OR *the strength of the recommendation changes*’ as management indications [27]. The guidance also provided management options for living CPG recommendations: no modification, modification of elements in the recommendation, merging recommendations, splitting recommendations, retirement, and removal [26]. With Table A13 and Figure A5 (Additional file 1) we adapted information found in living recommendation literature [18, 26, 27] for illustrative purposes to provide a hypothetical example of a living strategy.

### Considerations for variables

The needs and capabilities of an organization may be a factor in selecting detection and staging variables for a tailored maintenance strategy. However, literature may also provide some evidence about which variables to use. One study reported that both the ‘*number of new trials*’ and the ‘*identification of new drugs*’ were predictors for the decision to update SRs in a multivariable model [28]. The authors reported that ‘*a newly approved indication for an existing drug*’ was not a significant predictor. Another study predicted the probability that conclusions would change in an update [25]. Three variables (i.e. *effect size ratio, I-squared, power*) were not significant predictors in univariable analyses. Six variables were significant predictors in univariable analyses while only the ‘*number of new trials*’ and the ‘*log weight ratio*’ remained in the multivariable model predicting changes in conclusions. The exclusion of the four other variables (i.e. *large new trial, log participant ratio, logit standard error, log study ratio*) in the multivariable analysis indicates that these variables carried less predictive information. Variables containing less predictive information might still be good enough as proxy variables when organizations are unable to spend their resources for obtaining data on the known best predictors.

### Considerations for tests

Different tests and test protocols may provide information with different predictive strength on the same variable. Surveying experts for new evidence is arguably less resource intensive than performing a systematic literature search and selection. However, a systematic approach of search and selection might yield higher predictive information in terms of the number of identified studies. Systematic searches and selections might not be feasible for resource-limited organizations. Especially when individual searches and manual literature selections are performed for every key question in the organization’s portfolio. This might change in the future when machine learning systems are deployed to reduce time investments [29, 30]. Nevertheless, the gained time investments from semi-automation currently might come at a loss of accuracy in the study selection [31, 32].

Even within a single test there could be a difference in the resulting predictive information as variations could arise in the test protocol. For example, a single-person literature screening and selection might result in more missed studies than an independent double-blind literature screening. Other examples of variations within literature search and selection protocols in favor of time efficiency can be found in rapid review methodology, where it is proposed to dual screen at least 20% of the abstracts [33]. Future considerations about the impact on the predictive quality of information in test protocols might include whether single or dual-person literature selections are assisted by machine learning systems. Currently, semi-automating the literature selection in a single person protocol could result in a larger risk of missing relevant literature in the selection [34].

### Considerations for monitoring

Conclusions and recommendations seem to get out of date at variable rates [1, 2], thus a prespecified time interval itself does not inform which specific SR or CPG needs maintenance. The function of a prespecified time interval in the POMBYTTS framework, rather, is to initiate a new cycle of (re)assessments. Cyclical monitoring can enable the detection of new developments, recurrences, and progression. To detect a recurrence, the target condition needs to be present again after previous management actions were initially carried out to resolve the presence of the target condition. However, in some circumstances the target condition may be present in the SR or CPG but is not severe enough to allocate resources to for further maintenance actions, such as updating. Cyclical monitoring could then be used to monitor the progression of the target condition over time until the threshold is reached and indications for the management option are present. For example, when new evidence is available and does warrant new recommendations or a change the direction or strength of the recommendation. Here, the target condition can be present but no indications for updating are present. Future reassessments may show that the threshold is reached, indicating an update is appropriate. Setting an appropriate time interval between reassessments could be difficult. The interval should be long enough for the target condition to develop or progress but short enough to do no excessive harm when the target condition already developed or progressed early. A living CPG concerning pharmacological interventions for neuropathic pain after spinal cord injury searched for new evidence after 21 months and 10 months thereafter, respectively [27]. The living SR [35] in the World Health Organization’s ‘*Therapeutics and COVID-19’* guideline [36] monitored the literature daily. The interval may be dependent on the rate of developments in the specific field, available resources, or urgency.

### Limitations

One limitation of the presented framework is that it remains theoretical and has not yet been piloted in real-world situations for the development of SR and CPG maintenance strategies. While current updating and maintenance strategies can be mapped to the framework, its practical implementation and usability have not been tested. This is particularly relevant when dealing with very large portfolios, as monitoring the entire portfolio can be resource intensive. To address this challenge, one potential solution is to select less resource intensive tests that still provide an acceptable level of predictive information.

Another limitation pertains to the search and selection of the literature for our review. The search strategy primarily focused on identifying literature related to updating, and other maintenance options were not specifically targeted. Additionally, only literature that reported at least one indicator for the need for updating was included, potentially excluding literature solely reporting considerations for alternative management options. However, this limitation mainly affects the extent of examples provided and does not impact the fundamental concepts and elements of the framework.

Furthermore, subjective decisions were made during the selection of literature. For instance, some processes were categorized as need for updating processes rather than prioritization processes [16, 21, 25, 37]. The examples of variables, tests, and thresholds in Additional file 1 were based on our interpretation for elements in the framework and may not align with the intended use in the original publications.

### Future directions

In the future, there is potential for an evidence ecosystem to emerge, connecting the primary research community, the evidence synthesis community, the guideline developing community, and their stakeholders [38]. Processes within organizations participating in the ecosystem need to assure that exchangeable products and cocreated products are trustworthy. In our opinion, this is two-fold: trustworthy in terms of quality (due to rigorous development procedures) and trustworthy in terms of up-to-date products (due to rigorous portfolio maintenance strategies). The current theoretical POMBYTT framework might be a valuable tool to potentially design or adapt maintenance strategies for organizations in an evidence ecosystem to keep their SRs or CPGs up-to-date. This might particularly be important for resource-constrained organizations who face challenges in allocating resources for maintenance activities. In an ideal world, using the maintenance framework results in a strategy where the whole portfolio can enter a maintenance strategy and receive appropriate management actions by selecting less resource intensive tests. However, organizations with limited resources could also use priority-setting assessments to spend the available resources for maintenance on those SRs or CPGs with the highest priority. This requires new concepts to be introduced to the current theoretical POMBYTT framework.

The two hypothetical strategies designed with the framework (Tables A11-12 and Figures A3-4 in Additional file 1) and the living strategy derived from information from living recommendation literature [18, 26, 27] mapped to the framework’s elements (Table A13 and Figure A5 in Additional File 1) might demonstrate the framework’s potential applicability and relevance for maintenance practices. However, the POMBYTT framework has not undergone empirical validation in real practice. Therefore, future research could focus on potential application in research and practice by assessing the usability and feasibility of the POMBYTT framework for designing maintenance strategies and thereafter assessing the feasibility of the designed strategy for maintaining a portfolio or SRs or CPGs in the real-world. Research within the scope of the framework could focus on identifying detection and staging variables with acceptable predictive qualities given the resources available to obtain data on these variables. Artificial intelligence might enable the use of sensitive literature search strategies while relieving the workload associated with literature selections. Organizations may then choose to reallocate freed up resources to improve other test protocols that could provide better predictive information but are more resource intensive.

## Conclusions

The choices regarding variables, tests, test protocols, indications, management options, and monitoring when designing a maintenance strategy with the theoretical POMBYTT framework will have a direct impact on the resulting processes in the strategy. These elements aid in thinking about and being explicit about how the strategy operates when designing a maintenance strategy. For the resource-constrained organization it seems important to consider what result in acceptable predictive information about the presence or absence of the target condition and management indications while minimizing the resource investments. Understanding the components in the framework may also be helpful for stakeholders and end-users of SRs and CPGs to informally screen whether the SR or CPG is potentially still valid. Although the theoretical POMBYTT framework needs testing in the real world, it highlights important elements that should be explicitly considered when designing or adapting maintenance strategies. By taking these elements into account, organizations might potentially develop maintenance strategies related to their needs and context. Furthermore, the framework shows that there can be multiple management options available within a strategy, even when the target condition is absent. This highlights the importance of considering alternative management options beyond solely focusing on updating, probably offering greater flexibility in maintenance approaches.

### Supplementary Information

**Additional file 1. A1.1.** Review methods. **A1.1.1.** Search strategy. **A1.2.** Eligibility and literature selection. **A1.3.** Data extraction and data handling. **A1.4.** Data analysis. **Figure A1.** Flow diagram of the study selection. **Table A1.** Reasons for exclusion. **Table A2.** General characteristics of included studies. **Figure A2.** The Portfolio Maintenance by Test-Treatment framework with outlined test-treatment concepts. **Table A1.** Reasons for exclusion. **Table A2.** General characteristics of included studies. **Figure A2.** The Portfolio Maintenance by Test-Treatment framework with outlined test-treatment concepts. **Table A3.** Examples from the literature review which could be used as detection variables. **Table A4.** Examples from the literature review which could be used as detection tests. **Table A5.** Examples from the literature review which could be used as detection test thresholds. **Table A6.** Examples from the literature review which could be used as staging variables. **Table A7.** Examples from literature review which could be used as staging tests. **Table A8.** Examples from the literature review which could be used as staging thresholds. **Table A9.** Examples from the literature review which could be used as management indications. **Table A10.** Empty process description table. **Table A11.** Process description table of the example strategy in organization A. **Figure A3.** Process flow diagram of the example strategy in organization A (see Table A11). **Table A12.** Process description table of the example strategy in organization B. **Figure A4.** Process flow diagram of the example strategy in organization B (see Table A12). **Table A13.** A hypothetical example of a ‘living’ recommendations strategy. **Figure A5.** Process flow diagram of the hypothetical ‘living’ example strategy.

## Data Availability

All data generated or analyzed during this study are included in this published article and its supplementary information file.

## Abbreviations

CPG: Clinical Practice Guideline
POMBYTT: Portfolio Maintenance by Test-Treatment
SR: Systematic Review
