# Supplementary material for: Designing tailored maintenance strategies for systematic reviews and clinical practice guidelines using the Portfolio Maintenance by Test-Treatment (POMBYTT) framework
Source: BMC Med Res Methodol. 2024 Feb 2;24:29. doi: 10.1186/s12874-024-02155-z (PMC10835980; doi:10.1186/s12874-024-02155-z)
Supplement: Supplementary file 1 — Additional file 1. A1.1. Review methods. A1.1.1. Search strategy. A1.2. Eligibility and literature selection. A1.3. Data extraction and data handling. A1.4. Data analysis. Figure A1. Flow diagram of the study selection. Table A1. Reasons for exclusion. Table A2. General characteristics of included studies. Figure A2. The Portfolio Maintenance by Test-Treatment framework with outlined test-treatment concepts. Table A1. Reasons for exclusion. Table A2. General characteristics of included studies. Figure A2. The Portfolio Maintenance by Test-Treatment framework with outlined test-treatment concepts. Table A3. Examples from the literature review which could be used as detection variables. Table A4. Examples from the literature review which could be used as detection tests. Table A5. Examples from the literature review which could be used as detection test thresholds. Table A6. Examples from the literature review which could be used as staging variables. Table A7. Examples from literature review which could be used as staging tests. Table A8. Examples from the literature review which could be used as staging thresholds. Table A9. Examples from the literature review which could be used as management indications. Table A10. Empty process description table. Table A11. Process description table of the example strategy in organization A. Figure A3. Process flow diagram of the example strategy in organization A (see Table A11). Table A12. Process description table of the example strategy in organization B. Figure A4. Process flow diagram of the example strategy in organization B (see Table A12). Table A13. A hypothetical example of a ‘living’ recommendations strategy. Figure A5. Process flow diagram of the hypothetical ‘living’ example strategy. [file 12874_2024_2155_MOESM1_ESM.docx]

ADDITIONAL FILE 1

ADDITIONAL FILE 1 - INDEX

[A1.1 Review methods 3](#_Toc137732174)

[A1.1.1 Search strategy 3](#_Toc137732175)

[A1.2 Eligibility and literature selection 4](#_Toc137732176)

[A1.3 Data extraction and data handling 4](#_Toc137732177)

[A1.4 Data analysis 4](#_Toc137732178)

[Figure A1 – Flow diagram of the study selection 6](#_Toc137732179)

[Table A1 – Reasons for exclusion 7](#_Toc137732180)

[Table A2 – General characteristics of included studies 11](#_Toc137732181)

[Figure A2 – The Portfolio Maintenance by Test-Treatment framework with outlined test-treatment concepts 15](#_Toc137732182)

[Table A3 – Examples from the literature review which could be used as detection variables 16](#_Toc137732183)

[Table A4 – Examples from the literature review which could be used as detection tests 18](#_Toc137732184)

[Table A5 – Examples from the literature review which could be used as detection test thresholds 19](#_Toc137732185)

[Table A6 – Examples from the literature review which could be used as staging variables 19](#_Toc137732186)

[Table A7 – Examples from literature review which could be used as staging tests 21](#_Toc137732187)

[Table A8 – Examples from the literature review which could be used as staging thresholds 22](#_Toc137732188)

[Table A9 – Examples from the literature review which could be used as management indications 23](#_Toc137732189)

[Table A10 – Empty process description table 27](#_Toc137732190)

[Table A11 – Process description table of the example strategy in organization A 28](#_Toc137732191)

[Figure A3 – Process flow diagram of the example strategy in organization A (see Table A11) 29](#_Toc137732192)

[Table A12 – Process description table of the example strategy in organization B 30](#_Toc137732193)

[Figure A4 – Process flow diagram of the example strategy in organization B (see Table A12) 32](#_Toc137732194)

[Table A13 – A hypothetical example of a ‘living’ recommendations strategy 33](#_Toc137732195)

[Figure A5 – Process flow diagram of the hypothetical ‘living’ example strategy. 35](#_Toc137732196)

[Literature list 36](#_Toc137732197)

### A1.1 Review methods

A formal literature review protocol was not prepared a priori and the literature review was not registered.

### A1.1.1 Search strategy

We searched MEDLINE (via PubMed) for relevant published literature on 10 October 2018 which was updated on 29 April 2020. The search string contained keywords with synonyms and was constructed as follows: ((need for updating) OR (prioritizing)) AND (methods) AND (systematic review OR guideline). Additionally, PubMed Health (discontinued on 31 October 2018 [1]) was searched for relevant reports on 10 October 2018 with a similar search string. Hits from PubMed Health were limited via the search engine’s interface by using the ‘methods resources’ filter. The complete search string for PubMed and PubMed Health are available under subheadings *A1.1.1.1* and *A1.1.1.2*. We used a previously published SR to identify handbooks that considered updating CPGs [2]. If available, the latest version of a handbook was obtained through the organization’s website. All reference lists from the included journal articles, reports, and handbooks were hand searched for additional relevant literature.

#### A1.1.1.1 PubMed search string

((need[tiab] OR needs[tiab] OR needed[tiab] OR signal[tiab] OR signals[tiab] OR require[tiab] OR requires[tiab] OR required[tiab] OR detect[tiab] OR when[tiab]) AND (update[tiab] OR updates[tiab] OR updating[tiab] OR updated[tiab] OR "out of date"[tiab])) OR (prioritize[tiab] OR prioritise[tiab] OR prioritization[tiab] OR prioritisation[tiab] OR priority[tiab] OR priorities[tiab] OR prioritized[tiab] OR prioritised[tiab] OR prioritizing[tiab] OR prioritising[tiab]) AND ("Models, Theoretical"[Mesh] OR method[tiab] OR methods[tiab] OR methodology[tiab] OR methodologies[tiab] OR process[tiab] OR processes[tiab] OR surveillance[tiab] OR strategy[tiab] OR strategies[tiab] OR system[tiab] OR systems[tiab] OR approach[tiab] OR approaches[tiab] OR criteria[tiab] OR checklist[tiab] OR tool[tiab] OR content[tiab]) AND ("Review literature as topic"[mesh] OR "meta-analysis as topic"[mesh] OR "Practice guidelines as topic"[mesh] OR "guidelines as topic"[mesh] OR "Time factors"[mesh])

#### A1.1.1.2 PubMed Health search string

FILTER: METHODS RESOURCES

((need[tiab] OR needs[tiab] OR needed[tiab] OR signal[tiab] OR signals[tiab] OR require[tiab] OR requires[tiab] OR required[tiab] OR detect[tiab] OR when[tiab]) AND (update[tiab] OR updates[tiab] OR updating[tiab] OR updated[tiab] OR "out of date"[tiab])) OR (prioritize[tiab] OR prioritise[tiab] OR prioritization[tiab] OR prioritisation[tiab] OR priority[tiab] OR priorities[tiab] OR prioritized[tiab] OR prioritised[tiab] OR prioritizing[tiab] OR prioritising[tiab])

### A1.2 Eligibility and literature selection

Studies, reports, and handbooks that described at least one signal or consideration to assess the need for updating of SRs or (sections of) CPGs were included. We did not consider time thresholds (e.g. *“update after 3 years”*) as signals that inform decisions in the assessment of the need for updating. We believed that time thresholds immediately trigger an update without providing information about the actual outdatedness and therefore do not inform decisions in assessing the need for updating. Literature was excluded when they were non-healthcare related, conference abstracts, oral or poster presentations, or when literature could not be obtained. Obvious non-relevant hits regarding unrelated topics were excluded by one author (MSO). When in slightest doubt the reference was advanced to the title and abstract screening phase. The resulting hits were screened on title and abstract by two authors (MSO, RGE) blinded for each other’s decision. The reference was advanced to the full text selection phase when in doubt or disagreement. Full text articles, reports, and handbooks were read and selected by two authors (MSO, RGE) while blinded for each other’s decision. When no consensus could be reached or unclarity persisted after reading the full text, a third author (LH) was consulted for a final decision.

### A1.3 Data extraction and data handling

One author (MSO) extracted the data using a standardized data-extraction form. General characteristics such as publication year, author, organization, country and title were extracted and if the indicator was described for an SR or CPG. Signals and considerations specifying an organization, field, or profession were replaced with generalized terms. For example, “*New serious safety alert issued by the FDA or Health Canada*” [3], was generalized to “*New serious safety alert issued by federal organizations*”. Multiple considerations or signals presented in one sentence or as a cluster were separated when assumed that they could be independent of each other. For example, “*The nature and volume of new evidence*” [4] was separated into “*The nature of new evidence*” and “*The volume of new evidence*”. Extracted signals and considerations were cross-checked by another researcher.

### A1.4 Data analysis

We used NVivo for Windows (NVivo qualitative data analysis software; QSR International Pty Ltd. Version 12) to identify common themes among the extracted data. One author (MSO) performed the qualitative coding. The emerging domains were discussed with one author (LH). Changes as a result of the discussion were made without the use of NVIVO. Additional data extracted from papers identified in the updated search strategy were placed under the existing domains. The extracted data was thereafter characterized as detection variables, detection tests, detection thresholds, staging variables, staging tests, staging thresholds, or management indications at our own discretion.

**PubMed on 10 October 2018**

(n = 6303)

**PubMed Health on 10 October 2018**

(n = 143)

The methodology resources filter was used through PubMed Health’s search engine interface

**Retrieved guideline development handbooks in January 2019**

(n = 26)

One handbook was replaced by a newer handbook from a different organization on their website

**Guideline development handbooks**

Identified by Vernooij et al.

(n = 35)

*Unable to retrieve (n = 9)*

**Hits from PubMed**

10 October (n = 122)

29 April (n = 29)

**Hits from PubMed Health**

(n = 143)

*Excluded:*

*duplicate (n = 18)*

*Obvious non-relevant hits (n = 6163)*

**Hits from PubMed**

(n = 55)

**Hits from PubMed Health**

(n =14)

**Guideline development handbooks**

(n = 26)

*Excluded based on title and abstract:*

*PubMed (n = 96)*

*PubMed Health (n = 129)*

**Hits from PubMed**

(n = 20)

**Hits from PubMed Health**

(n = 6)

**Guideline development handbooks**

(n = 19)

*Excluded based on full text:*

*PubMed (n = 35)*

*PubMed Health (n = 8)*

*Guideline development handbooks (n = 7)*

**Total hits included**

(n = 54)

*Added from reference check*

*(n = 6)*

*Added from other sources*

*(n = 3)*

**PubMed from October 2018 to 29 April 2020**

Update (n = 652)

*Excluded:*

*duplicate (n = 1)*

*Obvious non-relevant hits (n = 622)*

#### Figure A1 – Flow diagram of the study selection. Handbooks were identified through a previously published systematic review by Vernooij et al. [2]

| *Table A1 – Reasons for exclusion* | | | |
| --- | --- | --- | --- |
| **First author / organization** | **Year** | **Title** | **Reason for exclusion** |
| Agencia d'avaluacio de technologia i recerca mediques |  | Guies de pràctica clínica. | Could not be found online |
| Akl | 2017 | The SPARK Tool to prioritize questions for systematic reviews in health policy and systems research: development and initial validation. | No need for updating indicators were found |
| Akl | 2017 | Living systematic reviews: 4. Living guideline recommendations | No need for updating indicators were found: refers to Chung 2012 and Shekelle 2014 for thresholds for when to update. |
| Alderson | 2014 | Median life span of a cohort of National Institute for Health and Care Excellence clinical guidelines was about 60 months | No need for updating indicators were found |
| Alonso-Coello | 2011 | The updating of clinical practice guidelines: insights from an international survey | No need for updating indicators described |
| American College of Chest Physicians |  | Evidence-based Guideline Development Process | No need for updating indicators were found |
| American College of Physicians | 2010 | The development of clinical practice guidelines and guidance statements of the American College of Physicians: summary of methods. | No need for updating indicators were found |
| American society of clinical oncology |  | American Society of Clinical Oncology Guideline Procedures Manual. | Could not be found online |
| Arzneimittelkommission der deutschen ärtzeschaft | 2011 | Leitfaden für die Erstellung von Therapieempfehlungen. | No need for updating indicators were found |
| Ärztliche Zentralstelle Qualitätssicherung |  | National Disease Management Guidelines: Method Report. | Could not be found online |
| Atkins | 2012 | Priority setting in guideline development: article 2 in Integrating and coordinating efforts in COPD guideline development. An official ATS/ERS workshop report | Seems to cover new topics |
| Bastian | 2011 | Choosing health technology assessment and systematic review topics: the development of priority-setting criteria for patients' and consumers' interests | Seems to cover new topics |
| Battista | 1995 | Setting priorities and selecting topics for clinical practice guidelines | Seems to cover new topics/guidelines |
| Bero | 2013 | The Cochrane Collaboration review prioritization projects show that a variety of approaches successfully identify high-priority topics | No need for updating indicators were found |
| Bundersärtzekammer |  | National Disease Management Guidelines. | Could not be found online |
| Burgers | 2012 | Adaptation, evaluation, and updating of guidelines: article 14 in Integrating and coordinating efforts in COPD guideline development. An official ATS/ERS workshop report | No need for updating indicators were found |
| Canadian thoracic society | 2007 | Canadian Thoracic Society: Presenting a new process for clinical practice guideline production. | No need for updating indicators were found |
| Centrul Naţional de Studii Medicina Familiei |  | Metodologie elaborarii ghidului de practica. | No need for updating indicators were found |
| Chalmers | 1993 | preparing and updating systematic reviews of randomized controlled trials of health care | No need for updating indicators were found |
| Clark | 2006 | From outdated to updated, keeping clinical guidelines valid | No original data |
| Dalal | 2012 | A Pilot Study Using Machine Learning and Domain Knowledge To Facilitate Comparative Effectiveness Review Updating [Internet] | No need for updating indicators were found |
| Domus Medical Flemish College of General Practitioners |  | Algemeen Stramien voor de Ontwikkeling van Aanbevelingen van Goede Medische Praktijkvoering. | Could not be found online |
| Doyle | 2005 | Global priority setting for Cochrane systematic reviews of health promotion and public health research | No need for updating indicators were found |
| Drug Commission of the German Medical Association | 2006 | Handbuch zur Entwicklung regionaler Leitlinien. | No need for updating indicators were found |
| Duodecim Finnish Medical Society |  | Submitted NHS Evidence Accreditation Application. | Could not be found online |
| Duodecim Medical Publications |  | Preface: What is Evidence-Based Medicine Guidelines. | Could not be found online |
| El-Harakeh | 2019 | Prioritization approaches in the development of health practice guidelines: a systematic review | No need for updating indicators were found |
| European Region Of The World Confederation For Physical Therapy |  | Framework for Clinical Guideline Development in Physiotherapy. | Could not be found online |
| French | 2005 | Investing in updating: how do conclusions change when Cochrane systematic reviews are updated? | No need for updating indicators were found |
| Gartlehner | 2004 | Assessing the need to update prevention guidelines: a comparison of two methods | No need for updating indicators were found |
| Handoll | 2013 | A framework for effective collaboration between specialist and broad-spectrum groups for delivering priority Cochrane reviews | Seems to cover new topics/No need for updating indicators were found |
| Hoomans | 2012 | Systematizing the Use of Value of Information Analysis in Prioritizing Systematic Reviews [Internet] | No need for updating indicators were found |
| Jiang | 2016 | Essential methods and procedures on how to develop and update clinical practice guidelines. | Unobtainable, doi: 10.3760/cma.j.issn.0376-2491.2016.04.004 |
| Jiang | 2019 | Guideline for [Clinical Guidelines Constitution/Amendment] in China | No need for updating indicators were found |
| Joanna Briggs Institute Synthesis Science Unit |  | Best Practice Information Sheet (BPIS) Procedures. | Could not be found online |
| Jones | 2013 | Success for a novel approach to priority setting in South Australian public dental clinics | Seems to cover health care priority setting |
| Kim | 2018 | Identifying and prioritizing topics for evidence-based geriatric nursing practice guidelines in Korea | No need for updating indicators were found |
| Land | 2017 | A five-step approach for stakeholder engagement in prioritisation and planning of environmental evidence syntheses | Not healthcare related |
| Manafo | 2018 | Patient and public engagement in priority setting: A systematic rapid review of the literature. | Priority setting in health research and health eco-system |
| Martinez Garcia | 2012 | Strategies for monitoring and updating clinical practice guidelines: a systematic review | No original data |
| Martinez Garcia | 2014 | Updated recommendations: an assessment of NICE clinical guidelines | No need for updating indicators were found |
| Martinez Garcia | 2017 | Methodological systematic review identifies major limitations in prioritization processes for updating | No original data |
| McClarey | 1999 | Identifying priorities for national clinical guidelines | Seems to cover new topics |
| Moher | 2008 | When and how to update systematic reviews | Duplicate (PubMed) / No original data |
| Moher | 2007 | A systematic review identified few methods and strategies describing when and how to update systematic reviews | No original data |
| Moher | 2008 | When and how to update systematic reviews | No original data |
| Nasser | 2013 | An equity lens can ensure an equity-oriented approach to agenda setting and priority setting of Cochrane Reviews | No need for updating indicators were found |
| Nasser | 2013 | Ensuring relevance for Cochrane reviews: evaluating processes and methods for prioritizing topics for Cochrane reviews | No need for updating indicators were found |
| Nast | 2019 | Prioritizing topics in guideline development: results of a two-phase online survey of dermatologist members of the EADV | No need for updating indicators were found |
| Neuman | 2014 | Durability of class I American College of Cardiology/American Heart Association clinical practice guideline recommendations | No need for updating indicators were found |
| New Zealand Guidelines Group |  | Handbook for the Preparation of Explicit Evidence-base Clinical Practice Guidelines. | Could not be found online |
| NICE | 2013 | Interim Process and Methods Guide for the Clinical Guideline Updates Using Standing Committees Pilot Programme 2013 [Internet] | No need for updating indicators were found |
| NICE | 2013 | Interim Process and Methods of the Highly Specialised Technologies Programme [Internet] | No need for updating indicators were found |
| NICE | 2014 | Interim Methods Guide for Developing Service Guidance 2014 [Internet] | No need for updating indicators were found |
| Olav Vandvik | 2014 | [A new generation of reliable clinical practice guidelines through MAGIC] | No need for updating indicators were found |
| Pieper | 2019 | [Increasing the efficiency of guideline production: a narrative review] | No original data |
| Reveiz | 2010 | Prioritization strategies in clinical practice guidelines development: a pilot study | No need for updating indicators were found |
| Robinson | 2015 | Integrating Bodies of Evidence: Existing Systematic Reviews and Primary Studies | No original data |
| Sampson | 2008 | Surveillance search techniques identified the need to update systematic reviews | No need for updating indicators were found |
| Sanabria | 2020 | Prioritizing clinical guideline questions for updating: the UpPriority Tool | No need for updating indicators were found |
| Scott | 2018 | Cochrane acute respiratory infections group's stakeholder engagement project identified systematic review priority areas | No need for updating indicators were found |
| Sibbald | 2009 | Priority setting: what constitutes success? A conceptual framework for successful priority setting | No need for updating indicators were found |
| Stead | 2001 | Updating a systematic review – what difference did it make? Case study of nicotine replacement therapy | No need for updating indicators were found |
| Synnot | 2019 | Selecting, refining and identifying priority Cochrane Reviews in health communication and participation in partnership with consumers and other stakeholders | No need for updating indicators were found |
| Taylor | 2019 | Use of multi-attribute decision-making to inform prioritization of Cochrane review topics relevant to rehabilitation | No need for updating indicators were found |
| Therapeutic Guidelines Limited | 2017 | How Therapeutic Guidelines are produced. | No need for updating indicators were found |
| Tsertsvadze | 2011 | Updating Comparative Effectiveness Reviews: Current Efforts in AHRQ’s Effective Health Care Program | Duplicate (PubMed) / No original data |
| Tsertsvadze | 2011 | Updating comparative effectiveness reviews: current efforts in AHRQ's Effective Health Care Program | No original data |
| Tugwell | 2013 | Methods for setting priorities in systematic reviews | No need for updating indicators were found |
| Vernooij | 2014 | Guidance for updating clinical practice guidelines: a systematic review of methodological handbooks | No original data |
| Voisin | 2008 | Strategies in assessing the need for updating evidence-based guidelines for six clinical topics: an exploration of two search methodologies | Concerned search methodologies |
| Wale | 2013 | The Cochrane Library review titles that are important to users of health care, a Cochrane Consumer Network project | No need for updating indicators were found |
| Zarnke | 2000 | A novel process for updating recommendations for managing hypertension: rationale and methods | Unobtainable, could not be found on the journal's website |

| *Table A2 – General characteristics of included studies* | | | |
| --- | --- | --- | --- |
| **Author or organization**^†^ | **Year** | **Country**^‡^ | **Brief description of the need for updating process** |
| Agbassi et al. [5] | 2014 | Canada | A questionnaire with criteria categorizes guidelines in four categories: endorse, defer, review, archive. Guidelines are assessed by a methodologist and a clinical expert using the questionnaire. Guidelines flagged as ‘review’ were assessed by another questionnaire, which resulted in a decision: endorse, update, or archive. |
| Agency for Health Care Policy and Research [6] | 1994 | USA | A public meeting to address the need for updating and the timing of an update is held as soon as sufficient data is obtained that indicates an update may be needed. |
| Ahmadzai et al. [7] | 2013 | Canada | After a focused search, qualitative and quantitative signals were assessed. Experts could indicate if the conclusions were still valid and could provide any new references that might invalidate conclusions. Experts could also provide any references that were important to the topic, but did not invalidate conclusions. Safety alerts were assessed. Evidence was combined with expert opinion and conclusions were categorized: up-to-date, possibly out-of-date, probably out-of-date, or out-of-date. |
| American Academy of Orthopaedic Surgeons [8] | 2011 | USA | Guidelines that are at least five years old are listed. A committee makes the decision whether to update or not based on criteria. |
| American College of Cardiology Foundation and American Heart Association [9] | 2010 | USA | A research analyst and the chair compare current recommendations against the latest data. The writing committee is surveyed whether (parts of) a guideline needs updating. Late-breaking trials were reviewed and regulatory bodies were monitored as well. The necessity of a guideline review is then determined. |
| American College of Occupational and Environmental Medicine [10] | 2017 | USA | Literature reviews were performed periodically to identify major changes in the literature. Major changes would require more frequent focused updates. |
| American urological association [11] | 2015 | USA | A systematic review of literature published since the release of the guideline is performed. A panel determines whether a limited or full revision is warranted. |
| Barrowman et al. [12] | 2003 | Canada | A ‘diagnostic’ test was performed for whether meta-analyses were out of date based on the cut-off of the new participant ratio being greater than one. |
| Bashir et al. [13] | 2018 | Australia | Signals were assessed retrospectively in a cohort of updated systematic reviews. Literature included in the updated systematic review and trial registries were assessed to obtain updating signals. |
| Becker et al. [14] | 2014 | Germany | A systematic monitoring of red flags, information and commentaries by the guideline authors, guideline users or experts, and relevant evidence was described. Additional searches could be performed when necessary, followed by a final decision whether an update was indicated. |
| Becker et al. [15] | 2018 | Germany | A limited search was performed and new potentially relevant evidence was reported. The report was sent to the guideline group and an online survey was conducted. Results were summarized and a consensus meeting was held to determine which sections were in high need for updating.  A multidisciplinary working group answered a set of questions in a survey. The survey results were analyzed and debated to determine which guidelines needed updating. |
| Chung et al. [16] | 2012 | USA | Two methods were used. The modified Ottawa-method used a focused literature search. New evidence was screened for their relevance to the review conclusions and qualitative, quantitative and ‘other’ indicators were assessed. The RAND-method used a limited literature search. Field experts were asked whether conclusions were still valid and to provide additional evidence. |
| Cumpston et al. [17] | 2019 | UK | A set of questions were answered sequentially. If any question was answered negatively, no update would be performed. Otherwise, an update would be performed. |
| Davis et al. [18] | 2007 | Canada | Persons familiar with the topic may conduct limited literature searches on a routine basis. The working group may identify literature that ensures a revision of the guideline. |
| Dumonceau et al. [19] | 2012 | Switzerland | NR^§^ |
| Garner et al. [20] | 2016 | UK | A set of questions were answered sequentially. If any question was answered negatively, no update would be performed. Otherwise, an update would be performed. |
| Garritty et al. [21] | 2010 | Canada | NF^§^ |
| Guidelines and Protocols Advisory Committee [22] | 2017 | Canada | NF^§^ |
| Haller et al. [23] | 2015 | Belgium | NF^§^ |
| Haute Autorité de Santé [24] | 2016 | France | NF^§^ |
| Howell [25] | 2015 | Australia | A literature review from the date of the last search for the guideline is performed. Guideline writers are asked to appraise additional relevant studies and to determine whether recommendations may change (add or remove a recommendation, strength of a recommendation, and/or change a recommendation). |
| Iorio et al. [26] | 2009 | Italy | A coordinator may invite to draft an update when a relevant paper is published. |
| Javaher [27] | 2015 | USA | Epidemiologists review and revise. All potential changes and updates were presented to an advisory commission for their recommendations. |
| Johnston et al. [28] | 2003 | Canada | New evidence was searched. A working group was informed about the new evidence through a summary report and made a decision whether to update. |
| Kwaliteitsinstituut voor de Gezondheidszorg CBO [29] | 2007 | The Netherlands | Associations relevant to the clinical guideline are notified when data from several criteria shows that updates are important. Thereafter, it is decided whether an update is warranted. |
| Lyratzopoulos et al. [4] | 2012 | UK | Clinical experts could provide comments on whether there had been substantial changes in the evidence since the guideline was published. An updated search could be performed. The expert comments and the information retrieved by the literature search were discussed and a decision about updating the guideline was made. |
| Martínez García et al. [30] | 2017 | Spain | Each clinical question of the guideline was classified in one of the three categories: to be reviewed, valid, or new clinical question. |
| Martínez García et al. [31] | 2014 | Spain | New evidence that could change recommendations was gathered through expert identification and experts evaluated whether recommendations were still up to date. Furthermore, a literature search was performed. A decision for updating (i.e. valid or in need for updating) was made on references that could potentially trigger an update. |
| Meerhoff et al. [32] | 2016 | The Netherlands | Implementation problems are continuously monitored. Scientific expert from the previous working group inform about new literature since the guideline was published. The experts also keep informed about whether other organizations are planning to develop or update potentially relevant guidelines. Guideline experts advised the organization’s board about the need to update one or more guidelines. |
| Mickenautsch et al. [33] | 2013 | South Africa | A modified Ottawa-method was used where a basic literature search was conducted. The qualitative, quantitative, and ‘other’ signals were assessed and assigned after study selection. |
| Murad et al. [34] | 2015 | USA | Periodically monitoring the literature for new evidence. |
| National Health and Medical Research Council [35] | 2009 | Australia | A multidisciplinary group (similar to the guideline development group) should assess the guidelines whether new evidence should be incorporated. |
| National Institute for Health and Care Excellence [36] | 2014 | UK | Without the need for a formal check, the need to update recommendations was assessed on a case-by-case basis when there are safety concerns. Decisions to update a guideline are based on the cumulative assessment of the evidence published since the guideline publication. Every two years the guidelines are assessed. Less resource-intensive checks are performed at 2/6/10-year timepoints, while extensive checks are performed at 4/8-year timepoints. Findings of the checks are discussed. |
| National Institute for Health and Clinical Excellence [37] | 2013 | UK | New evidence, views of the guideline development group and additional information about the relevance of the guideline is assessed. A decision was made on the surveillance review proposal: substantial update, rapid update, no update, transfer to static list, withdraw. |
| Newberry et al. [38] | 2013 | USA | Two methods were used separately or combined. The Ottawa-method had a focused literature search. New evidence was screened for relevance for the review conclusions. Qualitative and quantitative indicators were assessed and assigned. The RAND-method had a limited search. Experts responded to a questionnaire and could indicate whether conclusions were still valid. Experts also could provide new evidence. The evidence from the literature search and the expert opinion were then assessed. |
| Pattanittum et al. [39] | 2012 | Thailand | Several methods were compared. All methods were of statistical nature (or used quantitative indicators) and had predefined cut-off values. Methods included the assessment of sufficiency and stability, changes in effect size, the new participant ratio, changes in statistical significance, and simulation-based power. |
| Peterson et al. [3] | 2011 | USA | Based on a majority vote informed by new evidence and any variety of factors in addition to the evidence the decisions to update were made. |
| Rosenfeld et al. [40] | 2013 | USA | Reviewers were asked to complete a summary grid and to review each key statement. Thereafter, reviewers suggested: keep as is, keep but modify, or discard. Experts could also identify new evidence or quality improvement opportunities. Additionally, a literature search should be performed. |
| Scottish Intercollegiate Guidelines Network [41] | 2015 | UK | Individuals that comment on guidelines were asked to develop a small change proposal. The advisory group assessed small proposals and small changes may be agreed upon. The advisory group will also consider whether new evidence warrants a selected update or full review. A full proposal is then assessed together with new topics. |
| Shekelle et al. [42] | 2014 | USA | Literature was searched in databases and top-rated medical journals (general and topic specific specialty journals). Experts were asked to comment on whether the conclusion were out of date. Both the new evidence and expert opinions were assessed to determine whether it indicated a need for updating. |
| Shekelle et al. [43] | 2001 | USA | Experts were asked if there was new evidence and whether the new evidence was sufficient for an update. Experts were also asked whether there were new guideline statements (within the original scope) to be added. After that, a limited literature search was performed. Literature and expert opinion were reviewed for a final decision about the magnitude of an update: major update, minor update, or still valid. |
| Shekelle et al. [44] | 2009 | USA | A limited literature search was performed. Experts responded to a questionnaire and could indicate whether conclusions were still valid. Experts also could provide new evidence. The evidence from the literature search and the expert opinion were then assessed. |
| Shekelle et al. [45] | 2014 | USA | A limited search was performed. Experts responded to a questionnaire and could indicate whether conclusions were still valid. Experts also could provide new evidence. The evidence from the literature search and the expert opinion were then assessed. |
| Shekelle et al. [46] | 2011 | USA | Two methods were used. The Ottawa-method had a focused literature search. New evidence was screened for relevance for the review conclusions. Qualitative and quantitative indicators were assessed and assigned. The RAND-method had a limited search. Experts responded to a questionnaire and could indicate whether conclusions were still valid. Experts also could provide new evidence. The evidence from the literature search and the expert opinion were then assessed. |
| Shekelle et al. [47] | 2001 | USA | A limited search is proposed. Guideline recommendations are assessed by experts. Experts may indicate whether new recommendations should be present and whether they are aware of new evidence. An expert panel decides whether the guideline recommendation needs updating. |
| Shojania et al. [48] | 2007 | Canada | Literature was searched for systematic reviews and trials. Qualitative and quantitative indicators were assigned. Qualitative signals had two levels of importance: potentially invalidating, or major changes. A discussion was held for additional searches and the updating status was finalized. |
| Shojania et al. [49] | 2007 | Canada | A search for new systematic reviews and trials on the specific topic was performed. Candidate trials were screened and eligible studies were added to the existing meta-analysis. Qualitative and quantitative signals were assessed. An additional search could be performed when needed and the updating status was finalized. |
| Soll et al. [50] | 2008 | USA | Editors and regional coordinators assessed the need for updating based on criteria. |
| Sutton et al. [51] | 2009 | UK | One method used the new participants ratio, which indicates the number of new participants in the (null) meta-analysis to obtain significance. A second method was an adjusted power calculation. Here, simulations were used to calculate the proportion of simulations where the null hypothesis was rejected. Other indicators can be used in the simulation-based framework as well. |
| Takwoingi et al. [52] | 2013 | UK | A multi-component tool was used. First it was assessed whether the clinical question was already answered by the available evidence and whether it was still relevant. Then it was considered whether there were any new factors relevant to the review and it was assessed whether there were new studies. Availability of a review team was taken into account. Following a flow-chart, the decision could be made: to update now, to be updated, or to not update just yet. |
| US Preventive Task Force [53] | 2015 | USA | Based on indicators, a brief background paper was written. The paper was discussed and each topic was labeled as ‘active’ or ‘inactive’. |
| Welsh et al. [54] | 2015 | UK | First it was assessed whether the clinical question already was still relevant. Then it was considered whether there were any new factors relevant to the review. It was also assessed whether there were new studies and whether they were likely to impact the review conclusions. Availability of a review team was taken into account. Following a flow-chart, the decision could be made: to update now, to be updated, or to not update just yet. |
| Working Group for CPG Updates [55] | 2010 | Spain | A limited literature search was performed. Guideline developers or experts were asked whether there was new relevant literature or new recommendations should be added. Furthermore, user needs and guideline context were assessed. A decision about whether or not to update was made based on the provided data. |
| World Health Organization [56] | 2014 | Switzerland | A continual assessment of how new information may affect recommendations in a rapid advice guideline. For recommendations that may be out of date in guidelines (based on new evidence), implementers and stakeholders are made aware of the uncertainty and plans for updating the recommendations. |
| *^†^The first author or organization that was described was extracted.*  *^‡^The country mentioned in the first affiliation of the first author, or the country of the organization was extracted*  *^§^Not found or could not be deduced.*  *RAND: Research ANd Development, from the RAND Corporation*  *UK: United Kingdom*  *USA: United States of America* | | | |

***Figure A2 – The Portfolio Maintenance by Test-Treatment framework with outlined test-treatment concepts: diagnosis, staging, management, and monitoring (outlined in blue). The magenta line schematically represents the cyclical nature of monitoring. The dotted part of the magenta line represents the (re)assessments in the maintenance strategy after a prespecified time interval.*

| *Table A3 – Examples from the literature review which could be used as detection variables* | | | |  |
| --- | --- | --- | --- | --- |
| **Domain** | **Item** | **Reference** | **Described for** | |
| *New evidence* | Emerging scientific evidence | [27] | Guideline | |
|  | Publication in a peer reviewed journal | [9] | Guideline | |
|  | Significant new clinical trials and/or peer reviewed literature on the topic | [9] | Guideline | |
|  | Nonrandomized data deemed important on the basis of results impacting safety and efficacy assumptions | [9] | Guideline | |
|  | Relevant paper published in the intervening period | [26] | Guideline | |
|  | Are participants of the multidisciplinary guideline working group aware of new potentially relevant evidence that was not identified by the limited search? | [15] | Guideline | |
|  | New evidence | [34] | Guideline | |
|  | Whether there is any new evidence that should be incorporated | [35] | Guideline | |
|  | New relevant evidence since the guideline publication | [36] | Guideline | |
|  | Abstracts of primary or secondary evidence that has been published since the end of the search period for the guideline, with critical appraisal of key papers | [36] | Guideline | |
|  | Information on important new evidence in the field | [41] | Guideline | |
|  | Has new evidence emerged since the original clinical practice guideline was drafted? | [55] | Guideline | |
|  | The updating of recommendations should be considered in light of data published in the scientific literature or significant changes in practice since the publication of the recommendations [Translated from French: L’actualisation des recommandations doit être envisagée en fonction des données publiées dans la littérature scientifique ou des modifications de pratique significatives survenues depuis la publication des recommandations] | [24] | Guideline | |
|  | Comments from the public regarding new scientific evidence or new technologies that may warrant the updating of a clinical practice guideline | [6] | Guideline | |
|  | Changes in the evidence that current practice is optimal | [40, 43, 47] | Guideline | |
|  | Changes in evidence on existing benefits and harms of interventions | [18, 22, 40, 47] | Guideline | |
|  | Changes in the evidence on the benefits and harms of existing interventions | [43] | Guideline | |
|  | In case of new evidence | [23] |  | |
|  | The publication of a study that was included in the update of primary outcome meta-analysis within a year of the systematic review being published† | [13] | Systematic review | |
|  | The completion date of a study that was included in the update of primary outcome meta-analysis within a year of the systematic review† | [13] | Systematic review | |
|  | New information that is now available | [50] | Systematic review | |
|  | Missing information that is now available | [50] | Systematic review | |
|  | Information from existing studies (e.g. information about new treatment regimens, population subgroups, harms, economic data, or outcome measures, including data from ongoing studies or previously missing data) | [52] | Systematic review | |
|  | Additional information from existing studies | [54] | Systematic review | |
|  | Potentially relevant evidence from (limited) literature searches (if necessary, additional searches could be conducted) | [14] | Systematic review | |
|  | Are there any new studies, or new information? | [20] | Systematic review | |
|  | Reporting of serious or ‘new’ serious adverse events | [21] | Systematic review | |
|  | Identification of newly approved drug | [3] | Systematic review | |
|  | Identification of newly approved indication for previously included drug | [3] | Systematic review | |
| *Changes to the procedures, methods, resources, or contents* | Changes in the available interventions | [18, 22, 40, 43, 47] | Guideline | |
|  | Changes in the outcomes that are considered important | [18, 22, 40, 43, 47] | Guideline | |
|  | Changes in the values placed on outcomes | [40, 43, 47] | Guideline | |
|  | Expanding or narrowing of the scope of the guideline [Translated from Dutch: Uitbreiding of inkrimping van de afbakening van de richtlijn] | [29] | Guideline | |
|  | Should the structure or the scope of the clinical practice guideline alter? | [15] | Guideline | |
|  | Are there new relevant subject areas which are not considered to date? | [15] | Guideline | |
|  | Do the questions and search criteria as they are in the document address current needs, such that an updated literature search would be useful and identify relevant evidence? | [5] | Guideline | |
|  | changes in health technologies | [27] | Guideline | |
|  | changes in procedures | [27] | Guideline | |
|  | Changes in the current practice [Translated from Dutch: Veranderingen in de huidige praktijk] | [29] | Guideline | |
|  | Changes in the resources available for health care | [18, 22, 40, 43, 47] | Guideline | |
|  | Has there been a sudden increase in costs and utilization? | [27] | Guideline | |
|  | New methodology (e.g. new statistical techniques, or changes is methodological guidance) | [52] | Systematic review | |
|  | New changes in methodology | [54] | Systematic review | |
|  | Are there any new relevant methods? | [20] | Systematic review | |
|  | New inclusion criteria (outcomes; interventions; populations, methodological advances/new analysis) | [21] | Systematic review | |
| *Alerts, feedback, requests, and comments* | Any other pertinent factors provided by the clinical experts | [4] | Guideline | |
|  | Drugs and medical devices alerts | [30] | Guideline | |
|  | Changes/announcements/policies on both existing and emerging areas of disease assessment and treatment | [9] | Guideline | |
|  | Comments from healthcare providers on the guideline [Translated from Dutch: Commentaar van zorgverleners op de richtlijn] | [29] | Guideline | |
|  | All comments received on the organization’s published guidelines | [41] | Guideline | |
|  | Request and requirements for review and update from the practice community, key stakeholders, and other sources of free relationships with industry or other potential bias | [9] | Guideline | |
|  | New serious safety alert issued by federal organizations | [3] | Systematic review | |
|  | Information and commentaries by the clinical practice guideline authors, other experts, and guideline users | [14] | Systematic review | |
|  | Alerts and information on medical product safety published by national authorities for medicine and current medical news published in medical newsletters | [14] | Systematic review | |
|  | Formal request from a policy or healthcare decision maker | [21] | Systematic review | |
|  | Response to user feedback | [52, 54] | Systematic review | |
| *Other* | Previous recommendation statement | [53] | Guideline | |
|  | Recommendations of other guideline developers | [53] | Guideline | |

| *Table A4 – Examples from the literature review which could be used as detection tests* | | | |
| --- | --- | --- | --- |
| **Domain** | **item** | **Reference** | **Described for** |
| *Expert and user solicitation* | Guidelines staff periodically assess whether an existing guideline remains current through the update literature review process | [11] | Guideline |
|  | Summary of brief literature search for new evidence | [53] | Guideline |
|  | Intelligence gathering on the perceived current relevance of the guideline, which may include responses to questionnaires, information on guideline and quality standard implementation, external enquiries about the guideline recommendations, internal intelligence (such as an organization’s guideline issues log), related guidance and quality standards (including placeholder statements in an organization’s quality standards), medicines licensing information, relevant national policy | [36] | Guideline |
|  | User perceptions: are any of the strategies or elements of the clinical practice guideline recommendations invalid? | [55] | Guideline |
|  | Opinions expressed by experts and by clinical practice guideline authors: are you aware of new evidence relevant to the clinical practice guideline recommendations? | [55] | Guideline |
|  | Context analysis: have there been any changes in the healthcare context of the clinical practice guideline? | [55] | Guideline |
|  | Context analysis: are there any strategies or elements of the clinical practice guideline recommendations invalid? | [55] | Guideline |
| *Traits of evidence* | m/n as the “new participant ratio” | [12] | Systematic review |
|  | The calculated power minus the proportion of significant results in the 10.000 iterations of re-meta-analyses | [39] | Systematic review |
|  | The participant ratio (q) was calculated from q =m/n where m is the observed number of participants in the study(ies) published within the most recent 3 years, and n is the expected number of participants in the study(ies) published within the most recent 3 years | [39] | Systematic review |
|  | Barrowman’s n new participant ratio | [52] | Systematic review |
|  | Participant ratio where the total number of participants (i.e. the total number in both the new and old studies) is compared to the total number in the old out-of-date meta-analysis | [52] | Systematic review |
|  | Large new study where the total number of participants is greater than the total number in any of the studies in the out-of-date meta-analysis | [52] | Systematic review |
|  | New pivotal study where the sample size is n-times that of any of the previous studies | [52] | Systematic review |
|  | Standard error ratio where the standard error of the new effect size from the updated meta-analysis is compared to the standard error from the out-of-date meta-analysis | [52] | Systematic review |
|  | Weight ratio where the total weight of the new studies is compared to the total weight of the old studies in the updated meta-analysis | [52] | Systematic review |
|  | Effect size ratio whether the effect size in the updated meta-analysis is compared to the effect size in the out-of-date meta-analysis | [52] | Systematic review |

| *Table A5 – Examples from the literature review which could be used as detection test thresholds* | | | |
| --- | --- | --- | --- |
| **Domain** | **Item** | **Reference** | **Described for** |
| New evidence | Recommendations were classified as still valid without key references. Key references were those that could potentially trigger an update. | [31] | Guideline |
|  | If new evidence is published (For recommendations that may be out of date) | [56] | Guideline |
|  | No new evidence or only confirmatory evidence and all responding experts assessed the conclusion as still valid [still up-to-date] | [7, 16, 38, 42, 44-46] | Systematic review |
| Traits of evidence | m/n>1 means a meta-analysis is out of date (for test: m/n as the “new participant ratio”) | [12] | Systematic review |
|  | Had to be ≥ 80% (for test: the calculated power minus the proportion of significant results in the 10.000 iterations of re-meta-analyses) | [39] | Systematic review |
|  | q>1 (for test: The participant ratio (q) was calculated from q =m/n where m is the observed number of participants in the study(ies) published within the most recent 3 years, and n is the expected number of participants in the study(ies) published within the most recent 3 years) | [39] | Systematic review |
|  | Ratio ≥ 5 (for test: Barrowman’s n new participant ratio) | [52] | Systematic review |
|  | Ratio ≥ 1.5 (for test: participant ratio where the total number of participants (i.e. the total number in both the new and old studies) is compared to the total number in the old out-of-date meta-analysis) | [52] | Systematic review |
|  | Yes / no (for test: large new study where the total number of participants is greater than the total number in any of the studies in the out-of-date meta-analysis) | [52] | Systematic review |
|  | n ≥ 3 (for test: new pivotal study where the sample size is n-times that of any of the previous studies) | [52] | Systematic review |
|  | Ratio ≤ 0.5 (for test: standard error ratio where the standard error of the new effect size from the updated meta-analysis is compared to the standard error from the out-of-date meta-analysis) | [52] | Systematic review |
|  | Ratio ≥ 1.5 (for test: Weight ratio where the total weight of the new studies is compared to the total weight of the old studies in the updated meta-analysis) | [52] | Systematic review |
|  | Absolute slope of the linear regression >0 (for stability) | [39] | Systematic review |
|  | Failsafe ratio >1 (for sufficiency) | [39] | Systematic review |
|  | Ratio ≥ 1.5, ratio ≤ 0.5, i.e. change of 50% (for test: effect size ratio whether the effect size in the updated meta-analysis is compared to the effect size in the out-of-date meta-analysis) | [52] | Systematic review |

| *Table A6 – Examples from the literature review which could be used as staging variables* | |  |  |
| --- | --- | --- | --- |
| **Domain** | **item** | **Reference** | **Described for** |
| Relevance | Is the document still relevant (clinically or to the care system as a whole in some way)? | [5] | Guideline |
|  | Relevance to prevention and primary care | [53] | Guideline |
|  | Did this guideline topic clarify practice or resolve an area of controversy? | [8] | Guideline |
|  | Estimate of disease burden | [53] | Guideline |
|  | Estimation of the clinical relevance | [15] | Guideline |
|  | Does the published review still address a current question? | [20] | Systematic review |
|  | The continuing importance of the review to decision makers | [17] | Systematic review |
| Traits of evidence | Strengths/weakness of research methodology and findings | [9] | Guideline |
|  | The likelihood that such information (i.e. the volume and quality of new evidence) would cause a change in the guideline's recommendation | [6] | Guideline |
|  | The availability of potentially relevant evidence that may influence the recommendations | [15] | Guideline |
|  | Do the current recommendations cover all relevant subjects addressed by the evidence, such that no new recommendations are necessary? | [5] | Guideline |
|  | Does newly identified evidence, on initial review, contradict the current recommendations, such that the current recommendations may cause harm or lead to unnecessary or improper treatment if followed? | [5] | Guideline |
|  | The volume of new evidence | [4, 6] | Guideline |
|  | The nature of new evidence | [4] | Guideline |
|  | The quality of new evidence | [6] | Guideline |
|  | Likelihood of additional studies influencing current findings | [9] | Guideline |
|  | New evidence that would significantly modify recommendations | [19] | Guideline |
|  | How much new evidence is available | [32] | Guideline |
|  | Does the strength of the original clinical practice guideline recommendations remain the same? | [55] | Guideline |
|  | Does this new information significantly affect recommendations? | [55] | Guideline |
|  | Is the newly published information likely to change the grade of recommendation in the existing guideline? | [8] | Guideline |
|  | Is the newly published information likely to reverse a recommendation? | [8] | Guideline |
|  | Do direct consequences arise from the new potentially relevant evidence (e.g. change of recommendation or new recommendation)? | [15] | Guideline |
|  | Large, randomized placebo-controlled trial(s) | [9] | Guideline |
|  | New scientific insights based on literature research [Translated from Dutch: Nieuwe wetenschappelijke inzichten, vast te stellen op basis van literatuuronderzoek] | [29] | Guideline |
|  | Number of participants in new studies | [21] | Systematic review |
|  | Change in width of 95% confidence interval | [49] | Systematic review |
|  | Change in statistical significance | [7, 16, 33, 38, 39, 46, 48, 49] | Systematic review |
|  | Change in effect size | [7, 16, 33, 38, 39, 46, 48, 49] | Systematic review |
|  | Change in clinical significance given specified limits of clinical equivalence | [52] | Systematic review |
|  | Will new studies/information/data change findings or credibility? | [20] | Systematic review |
|  | Number of new studies identified | [21] | Systematic review |
|  | Heterogeneity in the out of date meta-analysis based on tau squared | [52] | Systematic review |
|  | Number of new relevant trials | [3] | Systematic review |
|  | Months since completion of the original review or last full update | [3] | Systematic review |
|  | Totality (comprehensiveness) of all new evidence or data including harms & benefits | [21] | Systematic review |
| Access and inequality | Emergence of any evidence of inequality in access to services between different social groups that can be addressed through guideline recommendations | [41] | Guideline |
|  | Evidence of impacts on equality groups | [41] | Guideline |
|  | Extent to which the guideline is used in daily practice | [32] | Guideline |
|  | Has a review had a good access or use? | [20] |  |
| Resources | Changes in the restitution of diagnosis and treatment [Translated from Dutch: Veranderingen in de vergoeding van diagnostiek en behandeling] | [29] | Guideline |
|  | Changes in the available resources [Translated from Dutch: Verandering in de beschikbare middelen] | [29] | Guideline |
| Organizational decisions | Impact on current and/or likelihood of need to develop new performance measure(s) | [9] | Guideline |
|  | Seriousness of the problems or barriers to the implementation of the guideline | [32] | Guideline |
|  | Need for consistency with a new guideline or guideline revision | [9] | Guideline |
|  | Need for an internal organizational decision | [21] | Systematic review |
| Credibility | Time credibility | [21] | Systematic review |
|  | Will adoption of new methods change findings or credibility? | [20] | Systematic review |

| *Table A7 – Examples from literature review which could be used as staging tests* | |  |  |
| --- | --- | --- | --- |
| **Domain** | **Item** | **Reference** | **Described for** |
| Traits of evidence | Number of previous trials showing consistent results | [9] | Guideline |
|  | The ratio of the observed number of additional participants to the predicted number of additional participants to obtain statistical significance | [12] | Systematic review |
|  | The probability of producing statistically significant results when adding further studies (in which the results are consistent with those that already exist) to an existing meta-analysis | [51] | Systematic review |
|  | The number of additional subjects (on average) to obtain a statistically significant result from a null meta-analysis. (Barrowman’s n, the “new participant:n ratio”) | [51] | Systematic review |
|  | The ratio of the standard error of the predicted new estimate of effect to the existing effect | [51] | Systematic review |
|  | The ratio of the sum of weights allotted to the predicted new and existing studies in the updated meta-analysis | [51] | Systematic review |
|  | Study ratio where the total number of studies in the updated meta-analysis is compared to the total number of new studies. | [52] | Systematic review |
|  | Estimated probability of conclusions changing after the addition of new studies to an existing meta-analysis, by using: estimated p = invlogit(0.1207 + 0.4101 x weight ratio + 0.1836 x number of new trials) | [52] | Systematic review |
|  | Increase in number of patients | [49] | Systematic review |
|  | The likelihood of the effect size of the updated meta-analysis to lay inside, outside, or across prespecified limits representing clinical equivalence | [51] | Systematic review |
| Expert and user solicitation | Opinions expressed by experts and by clinical practice guideline authors: are there results which were once considered important but no longer? | [55] | Guideline |
|  | Opinions expressed by experts and by clinical practice guideline authors: should any diagnostic or treatment procedures be suspended or replaced by other procedures? | [55] | Guideline |
|  | User perceptions: have any treatment preferences/consequences been identified in connection with the clinical practice guideline recommendation? | [55] | Guideline |
|  | Opinions expressed by experts and by clinical practice guideline authors: should new recommendations within the scope of the original clinical practice guideline be included? | [55] | Guideline |
|  | Other sources of information on the continued relevance of the guideline | [36] | Guideline |
|  | The views of the Committee and topic experts. | [36] | Guideline |
|  | Opinions expressed by experts and by clinical practice guideline authors: does the new evidence alter the risk/benefit ratio? | [55] | Guideline |
|  | Opinions expressed by experts and by clinical practice guideline authors: is the new evidence significant enough to invalidate CPG recommendations? | [55] | Guideline |
|  | Identification and assessment of new evidence: have literature limited searches identified new evidence that invalidates the CPG recommendations? | [55] | Guideline |
|  | Opinions expressed by experts and by clinical practice guideline authors: are there any data showing that clinical practice is appropriate and the CPG is no longer necessary? | [55] | Guideline |
| Other | Data from visitations, performance measures, or other registries [Translated from Dutch: Gegevens uit visitatie, indicatoren of andere registraties] | [29] | Guideline |
|  | Other information relevant to the updating of guidelines may be obtained from evaluation studies conducted to examine the implementation of effects of the guideline; from development and use of guideline-derived medical review criteria, performance measures, and standards of quality; or from other related activities. | [6] | Guideline |

| *Table A8 – Examples from the literature review which could be used as staging thresholds* | |  |  |
| --- | --- | --- | --- |
| **Domain** | **Item** | **Reference** | **Described for** |
| Traits of evidence | Trial with sample size at least 3 times the size of previous largest trial | [49] | Systematic review |
|  | A new study with at least three times the number of participants as in previous studies | [16, 33, 46] | Systematic review |
|  | Change of at least 50% (for variable: change in width of 95% confidence interval) | [49] | Systematic review |
|  | Change of least 50% (for variable: change in effect size) | [7, 16, 33, 38, 39, 46, 48, 49] | Systematic review |
|  | New and old point estimates differ significantly | [48] | Systematic review |
|  | At least 50% (for test: increase in number of patients) | [49] | Systematic review |
|  | Change in statistical significance at a specified alpha level (alpha = 0.05) | [52] | Systematic review |
|  | A probability of around 50% is suggestive of the need to update the SR, but any threshold can be chosen (for test: Estimated probability of conclusions changing after the addition of new studies to an existing meta-analysis, by using: estimated p = invlogit(0.1207 + 0.4101 x weight ratio + 0.1836 x number of new trials)) | [52] | Systematic review |
| *Table A9 – Examples from the literature review which could be used as management indications* | | | |
| **Domain** | **Items** | **Reference** | **Described for** |
| Changes in the evidence | Major changes in the literature may necessitate more frequent updates | [10] | Guideline |
|  | Whether the new evidence provides data about specific uncertainties expressed in the original guidance | [4] | Guideline |
|  | Does new scientific literature indicate an update is needed? | [27] | Guideline |
|  | Potential key references (for a clinical question) that could potentially trigger an update | [30] | Guideline |
|  | Recommendations with one or more key references that could potentially trigger an update. | [31] | Guideline |
|  | Potentially invalidating changes in evidence due to: Opposing findings | [7, 16, 33, 38, 46, 48, 49] | Systematic review |
|  | Potentially invalidating changes in evidence due to: Substantial harm | [7, 16, 33, 38, 46, 48, 49] | Systematic review |
|  | Potentially invalidating changes in evidence due to: Superior new treatment | [7, 16, 33, 38, 46, 48, 49] | Systematic review |
|  | Major changes in evidence due to: Important changes in effectiveness short of ‘opposing findings’ | [7, 16, 33, 38, 46, 48, 49] | Systematic review |
|  | Major changes in evidence due to: Expansion of treatment | [48, 49] | Systematic review |
|  | Major changes in evidence due to: Clinically important expansion of treatment | [7, 16, 33, 38, 46] | Systematic review |
|  | Major changes in evidence due to: Important caveat | [48, 49] | Systematic review |
|  | Major changes in evidence due to: Clinically important expansion of treatment | [7, 16, 33, 38, 46] | Systematic review |
|  | Major changes in evidence due to: Opposing findings from discordant meta-analysis or non-pivotal trial | [7, 16, 38, 46, 48, 49] | Systematic review |
|  | Major changes in evidence due to: Opposing findings from discordant pivotal trial or systematic review/meta-analysis | [33] | Systematic review |
|  | New evidence in a situation where the original report had no evidence | [16, 33] | Systematic review |
|  | A major increase in the number of new studies | [16, 33, 46] | Systematic review |
|  | Pivotal trial, new meta-analysis, more recent practice guideline, or recent textbook does not contradict the previous review, but characterizes benefit in substantially different terms | [7, 38, 46] | Systematic review |
|  | Pivotal trial, meta-analysis including at least one new trial, practice guideline, recent textbook calls into question the use of the treatment on the basis of harm. A new result for harm that does not undermine use altogether but has clear potential to affect clinical decision making would count as a ‘major change’. | [48, 49] | Systematic review |
|  | A pivotal trial or systematic review (or guidelines) whose results called into question the use of the treatment based on evidence of harm or that did not proscribe use entirely but did potentially affect clinical decision-making. | [7, 16, 33, 38, 46, 48, 49] | Systematic review |
|  | A pivotal trial or meta-analysis (or guideline) whose results identified another treatment as significantly superior to the one evaluated in the original review, based on efficacy or harm. | [16, 33] | Systematic review |
|  | Pivotal trial, systematic review including at least one new trial, practice guideline, or recent textbook characterized another treatment as significantly superior to the one evaluated in the original meta-analysis (based on efficacy or harm)—to the point that it would be preferred in most settings. | [48, 49] | Systematic review |
|  | A pivotal trial or systematic review (or guidelines) whose results identified another treatment as significantly superior to the one evaluated in the original review, based on efficacy or harm. | [7, 38, 46] | Systematic review |
|  | A pivotal trial or meta-analysis (or guidelines) whose results identified another treatment as significantly superior to the one evaluated in the original review, based on efficacy or harm. | [16, 33] | Systematic review |
|  | Pivotal trial, new metaanalysis, more recent practice guideline, or recent textbook does not contradict the previous review, but characterizes benefit in substantially different terms | [46, 48, 49] | Systematic review |
|  | Pivotal trial, new meta-analysis, more recent practice guideline, or recent textbook has expanded of the role of the treatment | [48, 49] | Systematic review |
|  | Pivotal trial, new meta-analysis, more recent practice guideline, or recent textbook adds an important caveat, about the patient populations who benefit, way in which treatment has to be delivered in order to derive benefit, sustainability of benefit, or increases in harm that are not sufficient to undermine use altogether, but would clearly affect the decision to recommend treatment for at least some patient populations. | [48, 49] | Systematic review |
|  | The treatment has been characterized in sufficiently different terms to the cohort review that disagreement would have met criteria for ‘opposing findings’ except the source was not a pivotal trial, new meta-analysis, or more recent practice guideline, or recent textbook | [48, 49] | Systematic review |
| Impact on questions, findings, conclusions, or recommendations | A specific issue such as a new drug therapy or national issue such as a new government policy will give rise to a new key question | [41] | Guideline |
|  | The standard of care has shifted significantly since the last version of the document such that the questions only address the topic in part | [5] | Guideline |
|  | There are new significant options [for treatment, diagnosis, etc.] available that are not covered by the current questions, such that new questions would need to be added to the document | [5] | Guideline |
|  | New evidence relating to the topic that may warrant inclusion of additional recommendations. | [25] | Guideline |
|  | New evidence substantially changes a small number of recommendations in the guideline (corresponding to no more than two related key questions) | [41] | Guideline |
|  | The new evidence is inconsistent with the data used to inform the original practice guideline report. The strength of the new evidence will alter the conclusions of the original document. Recommendations in the original report will change. | [28] | Guideline |
|  | The new evidence is consistent with the data used to inform the original practice guideline report. The strength of the recommendation in the original report has been modified to reflect this additional evidence. | [28] | Guideline |
|  | Emerging data that suggests that the current recommendations need to be revised. (For rapid advice guidelines in case of a public health emergency) | [56] | Guideline |
|  | On initial review, does the newly identified evidence support the existing recommendation? | [5] | Guideline |
|  | New evidence relating to the topic that may warrant removal or change to a recommendation or suggestion | [25] | Guideline |
|  | New evidence relating to the topic that may alter the strength of a recommendation | [25] | Guideline |
|  | The availability of new methods that would have a meaningful impact on the review findings. | [17] | Systematic review |
|  | The availability of new data that would have a meaningful impact on the review findings. | [17] | Systematic review |
|  | Some new evidence that might change the comparative effectiveness review's conclusion, and /or a minority of responding experts assessed the review's conclusion as having new evidence that might change the conclusion | [7, 38, 42, 44-46] | Systematic review |
|  | Substantial new evidence that might change the comparative effectiveness review's conclusion, and/or a majority of responding experts assessed the review's conclusion as having new evidence that might change the conclusion | [7, 38, 42, 44-46] | Systematic review |
|  | New evidence that rendered the comparative effectiveness review's conclusion out of date or no longer applicable | [7, 38, 42, 44-46] | Systematic review |
| Organizational decisions | The revised guideline can be included in the short term in a multidisciplinary guideline/care standard | [32] | Guideline |
|  | The revised guideline may be important for positioning the health care discipline | [32] | Guideline |
|  | Did this guideline topic spur additional high-quality research from the specialty societies or the organization’s membership? | [8] | Guideline |
|  | Topic is otherwise outside the organization’s scope | [53] | Guideline |
|  | Inclusion in policy decision making or clinical practice guidelines (e.g. it might be important to update a review to include it in a new clinical guideline. If any such factors (termed updating signals) are identified, then a judgement is made on whether a signal for updating is likely or unlikely to change the results or conclusions of the review) | [52] | Systematic review |
|  | Inclusion in policy or clinical guidelines | [54] | Systematic review |
| Implementation | Evidence that the guideline is fully complied with by relevant organizations, and has become accepted practice | [41] | Guideline |
|  | There is evidence that the CPG has been fully implemented in the healthcare system and has been accepted as clinical practice | [55] | Guideline |
|  | Existing controversy or gap between evidence and practice | [53] | Guideline |
|  | Topic is not relevant to primary care provider because the service is not implemented in a primary care setting or not referable by a primary care provider | [53] | Guideline |
| Superseded or outdated | Not revised or amended following 10 years of publication | [11] | Guideline |
|  | The recommendations no longer apply, but the guideline is not of sufficiently high priority for updating. | [36] | Guideline |
|  | Topic is no longer relevant to clinical practice because of changes in technology, new understanding of disease etiology/natural history, or evolving natural history of the disease | [53] | Guideline |
|  | Discovery of a new preventive or treatment measures make the CPG obsolete | [55] | Guideline |
|  | The CPG recommendations are no longer applicable or are outdated | [55] | Guideline |
|  | Emergence of new treatments or preventive measures that render the guideline irrelevant | [41] | Guideline |
|  | Superseded by a more recent or more comprehensive guideline | [41] | Guideline |
|  | If a therapy was no longer favored, no longer in use, or in question because of a safety concern | [38] |  |
| Postpone | The new evidence is inconsistent with the data used to inform the original practice guideline report. However, the strength of the new evidence does not alter the conclusions of the original document. | [28] | Guideline |
|  | The new evidence is consistent with the data used to inform the original practice guideline report. The recommendations in the original report remain unchanged. | [28] | Guideline |
|  | Is there a good reason (e.g. new stronger evidence will be published soon, changes to current recommendations are trivial or address very limited situation) to postpone updating the guideline? | [5] | Guideline |
|  | Should full assessment and review of this document be deferred until next year? Consider yes if the document is less than three years old and there is no reason to doubt the recommendations | [5] | Guideline |
|  | Should full assessment and review of this document be deferred until next year? Consider yes if the document is between three and five years old and a justification can be provided as to why the recommendations can be considered trustworthy for another year | [5] | Guideline |
| Other | In general, if you believe that for the document to still be useful it will have to substantially be rewritten | [5] | Guideline |
|  | The document has been repeatedly deferred, and is now older than 5 years | [5] | Guideline |
|  | Guidelines that contain 50% or greater of its recommendations as consensus (expert opinion) or inconclusive generally should not be updated. | [8] | Guideline |
|  | Topic had a low public health burden | [53] | Guideline |
|  | The subject of another more recent CPG partly or wholly overlaps with that of the CPG | [55] | Guideline |
|  | Continued uncertainty regarding the study intervention | [50] | Systematic review |

| *Table A10 – Empty process description table* | | |
| --- | --- | --- |
| **Diagnosis** | *Target condition definition* | <definition> |
|  | *Detection variable* | <variable> |
|  | *Detection test (protocol)* | <test>  <protocol> |
|  | *Detection test threshold* | <threshold> |

|  | | **Target condition present** | **Target condition absent** |
| --- | --- | --- | --- |
| **Staging** | *Staging variable* | <variable> | <variable> |
|  | *Staging test (protocol)* | <test>  <protocol> | <test>  <protocol> |
|  | *Staging thresholds* | <thresholds> | <thresholds> |

|  | | **Target condition present** | **Target condition absent** |
| --- | --- | --- | --- |
| **Management** | *Management options (indications) and actions* | <option 1>  <indications>  <actions> | <option 1>  <indications>  <actions> |
|  |  | <option …>  <indications>  <actions> | <option …>  <indications>  <actions> |

| **Monitoring** | *Monitoring* | <timeframe> |
| --- | --- | --- |

| *Table A11 – Process description table of the example strategy in organization A* | | |
| --- | --- | --- |
| **Diagnosis** | *Target condition definition* | A CPG recommendation is outdated when new evidence is present |
|  | *Detection variable* | New peer-reviewed scientific evidence published since the publication of the CPG |
|  | *Detection test (protocol)* | A MEDLINE, EMBASE, and Cochrane Library search  (A repetition of the original MEDLINE, EMBASE, and Cochrane Library search is conducted. A double-blind title/abstract and full-text selection is performed based on the original selection criteria) |
|  | *Detection test threshold* | Any newly identified relevant peer-reviewed scientific evidence will result in the presence of the target condition |

|  | | **Target condition present** | | **Target condition absent** |
| --- | --- | --- | --- | --- |
| **Staging** | *Staging variable* | Likelihood of potential changes in the strength of the body of evidence *(target condition present)* | Number of new studies *(target condition present)* | Relevance for clinical practice (target condition absent) |
|  | *Staging test (protocol)* | Informal GRADEing of the body of evidence  (The new studies are added to the existing body of evidence. GRADE is informally used to assess the likelihood that the addition of new evidence results in a change of strength.) | A MEDLINE, EMBASE, and Cochrane Library search  (A repetition of the original MEDLINE and Cochrane Library search is conducted. A double-blind title/abstract and full-text selection is performed based on the original selection criteria. The number of selected studies is counted.) | A guideline panel vote  (The guideline panel is asked whether the recommendation is still relevant for clinical practice. The panel answers dichotomously [yes/no]. The proportion indicating that there is no longer any relevance is used for staging.) |
|  | *Staging thresholds* | Likely change of strength: Any probable change in the strength (according to the GRADE certainty of evidence)  No likely change of strength: No probable change in the strength (according to the GRADE certainty of evidence). | Low impact: 1 study  Moderate impact: 2-3 studies  High impact: ≥4 studies | Still relevant: 0-30% of the panel indicate that there is no relevance  Unsure: 30-60% of the panel indicate there is no relevance  No longer relevant: >60% of the panel indicate there is no relevance |

|  | | **Target condition present** | **Target condition absent** |
| --- | --- | --- | --- |
| **Management** | *Management options (indications) and actions* | Update (likely change of strength OR high impact of the number of new studies)  Actions: Initiate an update, inform guideline panel, allocate resources | Do not update, reassess at a later time point (still relevant or unsure)  Actions: Return the recommendation to the portfolio. Reassess at a later point in time. |
|  |  | Do not update, reassess at a later point in time (no likely change of strength)  Actions: Return the recommendation to the portfolio. Reassess at a later point in time. | Withdraw (no longer relevant)  Actions: Withdraw the recommendation from the guideline (and portfolio). Inform the guideline panel. Inform the end-users with a notice on the website. |

| **Monitoring** | *Monitoring* | The recommendations are assessed every year |
| --- | --- | --- |

### *Figure A3 – Process flow diagram of the example strategy in organization A (see Table A11)*

| *Table A12 – Process description table of the example strategy in organization B* | | | | |
| --- | --- | --- | --- | --- |
| **Diagnosis** | *Target condition definition* | A CPG recommendation is outdated when new interventions are available or when new evidence is present. | | |
|  | *Detection variable* | Availability of new intervention | Newly identified evidence | |
|  | *Detection test (protocol)* | Survey among experts to identify newly available interventions  (Experts are identified. A survey is prepared and sent to the experts. Experts can indicate whether they believe there are new intervention available.) | Survey among experts to identify newly available evidence  (Experts are identified. A survey is prepared and sent to the experts. Experts can suggest new evidence.) | A limited literature search in the top 5 general topic journals and top 5 specialty journals  (The top 5 general and specialty journals were ranked on impact factor. A specific search was conducted in MEDLINE, limited to the selected journals. Literature selection was performed by a methodologist.) |
|  | *Detection test threshold* | The identification of at least one new intervention will result in the presence of the target condition. Relevance is defined as being a preventive intervention resulting in the abundance of the current interventions or as an alternative to the current interventions. | Any new relevant evidence identified by the experts will result in the presence of the target condition. Relevance is defined by the original selection criteria. | Any new evidence identified from the literature search will result in the presence of the target condition. |

|  | | **Target condition present** | | | **Target condition absent** |
| --- | --- | --- | --- | --- | --- |
| **Staging** | *Staging variable* | Number of new relevant interventions | Relevance of newly identified interventions | Quality of new studies | Unwanted practice variation |
|  | *Staging test (protocol)* | Survey among experts to count newly available interventions  (Experts are identified. A survey is prepared and sent to the experts. Experts can indicate whether they believe there are new intervention available. Relevance is scored by the guideline panel.) | A survey for the guideline panel to assess relevance  (The guideline panel is consulted to assess the relevance of the identified interventions by the expert survey. Relevance is scored on a 7-point likert-scale: 0 = no relevante / 6 = highly relevant.) | Risk of bias assessment  (A methodologist assesses the risk of bias for newly identified potentially relevant RCTs with the Cochrane Risk of Bias Tool 2.0) | National data-registry analysis  (The recommendation is assessed according to the data from a national data-registry. Practice variation is assessed within the population.) |
|  | *Staging thresholds* | New interventions available: Any new intervention was identified  No new interventions available: No new intervention was identified | No relevance: A mean score of >1  Low relevance: A mean score of 1-3  Some relevance: A mean score of 3-5  High relevance: A mean score of >5 points | High quality studies available: At least one RCT with low risk of bias is available  Only low quality studies available: Only RTCs with high or unclear risk of bias are available | No variation: The recommendation is usually appropriately applied in practice indicating no further guidance is needed  No unwanted variation: There are signs that there are deviations from the recommendation in clinical practice that can be considered normal  Unwanted variation: There are signs that there are deviations from the recommendation in clinical practice that cause harm |

|  | | **Target condition present** | **Target condition absent** |
| --- | --- | --- | --- |
| **Management** | Management options (indications) and actions | Update ((new intervention available AND some or high relevance of the intervention) OR high quality study available OR unwanted practice variation)  Actions: Initiate an update, inform guideline panel, allocate resources | Do not update, reassess at a later point in time (no unwanted variation)  Actions: Return the recommendation to the portfolio. Reassess at a later point in time. |
|  |  | Do not update, reassess at a later point in time (No new relevant intervention available AND only low quality study available AND no variation or no unwanted variation)  Actions: Return the recommendation to the portfolio. Reassess at a later point in time.. | Archive (no variation)  Actions: Keep the recommendation on the website. Include a note that the recommendation is archived and is not to be updated. |
|  |  |  | Update (unwanted variation)  Actions: Initiate an update, inform guideline panel, allocate resources |

| **Monitoring** | *Monitoring* | The recommendations are assessed every two years. |
| --- | --- | --- |

### *Figure A4* – *Process flow diagram of the example strategy in organization B (see Table A12)*

| *Table A13 – A hypothetical example of a ‘living’ recommendations strategy based on information found in Akl et al. 2017 [57], El Mikati et al. 2022 [58], and Bragge et al. 2022 [59] which we adapted for illustrative purposes* | | |
| --- | --- | --- |
| **Diagnosis** | *Target condition definition* | The guideline recommendation is out of date when any new evidence becomes available |
|  | *Detection variable* | New scientific evidence according to the existing literature selection criteria |
|  | *Detection test (protocol)* | Literature search and selection  (MEDLINE and EMBASE are searched by rerunning the previous search strategy. The search strategy was developed by an information specialist. The title and abstract of the retrieved hits are screened by two methodologists independently. Conflicts are resolved. The resulting full text articles are screened by two methodologists for inclusion. Data is extracted from the Included articles and study quality is appraised.) |
|  | *Detection test threshold* | Any new relevant published evidence |

|  | | **Target condition present** | | **Target condition absent** |
| --- | --- | --- | --- | --- |
| **Staging** | *Staging variable* | Impact of new relevant published evidence on the recommendations | Impact of new relevant published evidence on the certainty of evidence | – |
|  | *Staging test (protocol)* | Expert panel discussion and voting  (The panel is supplied with the review question and guideline recommendation, with the new evidence and study quality, and with a set of questions to determine the influence of the new evidence on the recommendation. The panel discusses the possible implications in a physical meeting and votes whether the recommendation is impacted by the new evidence) | Certainty of evidence evaluation  (The GRADE Summary of Findings table is updated by methodologists with information from the new evidence and performing new GRADE assessments) | – |
|  | *Staging thresholds* | **Impacted:** when >75% of the panel indicates that the new evidence has impact on the recommendation  **Uncertain impact:** 25-75% of the panel indicates that the new evidence has impact on the recommendation  **No impact:** >25% of the panel indicates that the new evidence has impact on the recommendation | **High certainty:** when all critical outcomes for decision-making have a HIGH GRADE.  **Relatively certain:** when most, but not all, critical outcomes for decision-making have HIGH GRADE  **Moderate certainty:** when most or all critical outcomes for decision-making have MODERATE GRADE.  **Uncertain:** When most critical outcomes for decision-making have LOW or VERY LOW GRADE | – |

|  | | **Target condition present** | **Target condition absent** |
| --- | --- | --- | --- |
| **Management** | *Management options (indications) and actions* | **Update the recommendation and continue monitoring**  (when the expert panel indicates the recommendation is impacted by new evidence [>75%])  Perform an update using the regular procedures | **Do not update and continue monitoring**  (When no new relevant evidence was found)  Continue monitoring as defined under ‘Monitoring’ |
|  |  | **Do not update and continue monitoring**  (When the expert panel was uncertain [25-75%] OR indicated that there was no impact [<25%])  Continue monitoring as defined under ‘Monitoring’ | **–** |
|  |  | **Update the recommendation and retire from living status**  (when the expert panel indicates the recommendation is impacted by new evidence [>75%] AND the certainty of evidence became high)  Perform an update using the regular procedures and retire the recommendation from participating in this maintenance strategy. | – |

| **Monitoring** | *Monitoring* | Weekly reruns of the existing search strategy |
| --- | --- | --- |

### *Figure A5* – *Process flow diagram of the hypothetical ‘living’ example strategy (see Table A13)* *based on information found in Akl et al. 2017 [57], El Mikati et al. 2022 [58], and Bragge et al. 2022 [59] which we adapted for illustrative purposes.*

# Literature list

1. **PubMed Health to be discontinued October 31, 2018; content will continue to be available at NLM** [<https://ncbiinsights.ncbi.nlm.nih.gov/2018/07/31/pubmed-health-discontinued-october-2018-nlm/>]

2. Vernooij RW, Sanabria AJ, Solà I, Alonso-Coello P, Martínez García L: **Guidance for updating clinical practice guidelines: a systematic review of methodological handbooks**. *Implement Sci* 2014, **9**:3.

3. Peterson K, McDonagh MS, Fu R: **Decisions to update comparative drug effectiveness reviews vary based on type of new evidence**. *J Clin Epidemiol* 2011, **64**(9):977-984.

4. Lyratzopoulos G, Barnes S, Stegenga H, Peden S, Campbell B: **Updating clinical practice recommendations: is it worthwhile and when?** *Int J Technol Assess Health Care* 2012, **28**(1):29-35.

5. Agbassi C, Messersmith H, McNair S, Brouwers M: **Priority-based initiative for updating existing evidence-based clinical practice guidelines: the results of two iterations**. *J Clin Epidemiol* 2014, **67**(12):1335-1342.

6. Agency for Health Care Policy and Research: **Process for determining need for updates of clinical practice guidelines**. *Fed Regist* 1994, **59**(79 Pt 1):19723-19725.

7. Ahmadzai N, Newberry SJ, Maglione MA, Tsertsvadze A, Ansari MT, Hempel S, Motala A, Tsouros S, Schneider Chafen JJ, Shanman R *et al*: **A surveillance system to assess the need for updating systematic reviews**. *Syst Rev* 2013, **2**:104.

8. American Academy of Orthopaedic Surgeons Clinical Practice Guideline Unit: **Guideline Process Procedure for Updating Existing Evidence-Based Clinical Practice Guidelines**. In*.*, edn.: American Academy of Orthopaedic Surgeons; 2011.

9. American College of Cardiology Foundation and American Heart Association: **Methodology manual and policies from the ACCF/AHA Task Force on Practice Guidelines**. In*.*, edn.; 2010.

10. American College of Occupational and Environmental Medicine: **Methodology for ACOEM's Occupational Medicine Practice Guidelines - 2017 Revision**. In*.*, edn.; 2017.

11. American Urological Association: **American Urological Association Clinical Practice Guidelines Development**. In*.*, edn.; 2015.

12. Barrowman NJ, Fang M, Sampson M, Moher D: **Identifying null meta-analyses that are ripe for updating**. *BMC Med Res Methodol* 2003, **3**:13.

13. Bashir R, Surian D, Dunn AG: **Time-to-update of systematic reviews relative to the availability of new evidence**. *Syst Rev* 2018, **7**(1):195.

14. Becker M, Neugebauer EA, Eikermann M: **Partial updating of clinical practice guidelines often makes more sense than full updating: a systematic review on methods and the development of an updating procedure**. *J Clin Epidemiol* 2014, **67**(1):33-45.

15. Becker M, Jaschinski T, Eikermann M, Mathes T, Bühn S, Koppert W, Leffler A, Neugebauer E, Pieper D: **A systematic decision-making process on the need for updating clinical practice guidelines proved to be feasible in a pilot study**. *J Clin Epidemiol* 2018, **96**:101-109.

16. Chung M, Newberry SJ, Ansari MT, Yu WW, Wu H, Lee J, Suttorp M, Gaylor JM, Motala A, Moher D *et al*: **Two methods provide similar signals for the need to update systematic reviews**. *J Clin Epidemiol* 2012, **65**(6):660-668.

17. Cumpston M, Chandler J: **Chapter IV: Updating a review**. In: *Cochrane Handbook for Systematic Reviews of Interventions version 62 (updated February 2021).* edn. Edited by Higgins JPT, Thomas J, Chandler J, Cumpston M, Li T, Page MJ, Welch VA: Cochrane; 2021.

18. Davis DA, Goldman J, Palda VA, Canadian Medical Association: **Handbook on clinical practice guidelines**. Ottawa: Canadian Medical Association = Association médicale canadienne; 2007.

19. Dumonceau JM, Hassan C, Riphaus A, Ponchon T: **European Society of Gastrointestinal Endoscopy (ESGE) Guideline Development Policy**. *Endoscopy* 2012, **44**(6):626-629.

20. Garner P, Hopewell S, Chandler J, MacLehose H, Schünemann HJ, Akl EA, Beyene J, Chang S, Churchill R, Dearness K *et al*: **When and how to update systematic reviews: consensus and checklist**. *Bmj* 2016, **354**:i3507.

21. Garritty C, Tsertsvadze A, Tricco AC, Sampson M, Moher D: **Updating systematic reviews: an international survey**. *PLoS One* 2010, **5**(4):e9914.

22. Guidelines and Protocols Advisory Committee: **Guidelines and Protocols Advisory Committee Handbook: How our “Made in BC” Clinical Practice Guidelines and Protocols are Developed**. In*.*, edn.; 2017.

23. Haller MC, van der Veer SN, Nagler EV, Tomson C, Lewington A, Hemmelgarn BR, Gallagher M, Rocco M, Obrador G, Vanholder R *et al*: **A survey on the methodological processes and policies of renal guideline groups as a first step to harmonize renal guidelines**. *Nephrol Dial Transplant* 2015, **30**(7):1066-1074.

24. Haute Autorité de Santé: **Élaboration de recommandations de bonne pratique: Méthode Recommandations pour la pratique clinique**. In*.*, edn. Saint-Denis La Plaine: HAS; 2016.

25. Howell M: **KHA-CARI guidelines development manual**. In*.*, edn.; 2015.

26. Iorio A, Ageno W, Cosmi B, Imberti D, Lussana F, Siragusa S, Tormene D, Tosetto A, Cattaneo M: **Objectives and methodology: Guidelines of the Italian Society for Haemostasis and Thrombosis (SISET)**. *Thromb Res* 2009, **124**(5):e1-5.

27. Javaher SP: **Guideline Development Process in a Public Workers' Compensation System**. *Phys Med Rehabil Clin N Am* 2015, **26**(3):427-434.

28. Johnston ME, Brouwers MC, Browman GP: **Keeping cancer guidelines current: results of a comprehensive prospective literature monitoring strategy for twenty clinical practice guidelines**. *Int J Technol Assess Health Care* 2003, **19**(4):646-655.

29. Kwaliteitsinstituut voor de Gezondheidszorg CBO: **Evidence-based richtlijn ontwikkeling: Handleiding voor werkgroepen**. In*.*, edn.; 2007.

30. Martínez García L, Pardo-Hernández H, Sanabria AJ, Alonso-Coello P: **Continuous surveillance of a pregnancy clinical guideline: an early experience**. *Syst Rev* 2017, **6**(1):143.

31. Martínez García L, Sanabria AJ, García Alvarez E, Trujillo-Martín MM, Etxeandia-Ikobaltzeta I, Kotzeva A, Rigau D, Louro-González A, Barajas-Nava L, Díaz Del Campo P *et al*: **The validity of recommendations from clinical guidelines: a survival analysis**. *Cmaj* 2014, **186**(16):1211-1219.

32. Meerhof G, Heijblom K, Knoop J: **The Royal Dutch Society for Physical Therapy (KNGF) Guideline methodology manual 2016: Methodology for developing/updating and implementing KNGF guidelines**. In*.*, edn.; 2016.

33. Mickenautsch S, Yengopal V: **The modified Ottawa method to establish the update need of a systematic review: glass-ionomer versus resin sealants for caries prevention**. *J Appl Oral Sci* 2013, **21**(5):482-489.

34. Murad MH, Montori VM, Sidawy AN, Ascher E, Meissner MH, Chaikof EL, Gloviczki P: **Guideline methodology of the Society for Vascular Surgery including the experience with the GRADE framework**. *J Vasc Surg* 2011, **53**(5):1375-1380.

35. National Health and Medical Research Council: **A guide to the development, implementation, and evaluation of clinical practice guidelines**. 2009.

36. National Institute for Health and Care Excellence: **Developing NICE guidelines: the manual (April 2017 update)**. In*.*, edn.; 2014.

37. National Institute for Health and Care Excellence: **NICE Process and Methods Guides**. In: *Interim Clinical Guideline Surveillance Process and Methods Guide 2013.* edn. London: National Institute for Health and Care Excellence (NICE); 2013.

38. Newberry SJ, Ahmadzai N, Motala A, Tsertsvadze A, Maglione M, Ansari MT, Hempel S, Tsouros S, Schneider Chafen J, Shanman R *et al*: **AHRQ Methods for Effective Health Care**. In: *Surveillance and Identification of Signals for Updating Systematic Reviews: Implementation and Early Experience.* edn. Rockville (MD): Agency for Healthcare Research and Quality (US); 2013.

39. Pattanittum P, Laopaiboon M, Moher D, Lumbiganon P, Ngamjarus C: **A comparison of statistical methods for identifying out-of-date systematic reviews**. *PLoS One* 2012, **7**(11):e48894.

40. Rosenfeld RM, Shiffman RN, Robertson P: **Clinical Practice Guideline Development Manual, Third Edition: a quality-driven approach for translating evidence into action**. *Otolaryngol Head Neck Surg* 2013, **148**(1 Suppl):S1-55.

41. Scottish Intercollegiate Guidelines Network: **SIGN 50: a guideline developer's handbook**. In: *SIGN publication no 50.* edn. Edinburgh: SIGN; 2015.

42. Shekelle PG, Motala A, Johnsen B, Newberry SJ: **Assessment of a method to detect signals for updating systematic reviews**. *Syst Rev* 2014, **3**:13.

43. Shekelle PG, Ortiz E, Rhodes S, Morton SC, Eccles MP, Grimshaw JM, Woolf SH: **Validity of the Agency for Healthcare Research and Quality clinical practice guidelines: how quickly do guidelines become outdated?** *Jama* 2001, **286**(12):1461-1467.

44. Shekelle P, Newberry S, Maglione M, Shanman R, Johnsen B, Carter J, Motala A, Hulley B, Wang Z, Bravata D *et al*: **AHRQ Methods for Effective Health Care**. In: *Assessment of the Need to Update Comparative Effectiveness Reviews: Report of an Initial Rapid Program Assessment (2005–2009).* edn. Rockville (MD): Agency for Healthcare Research and Quality (US); 2009.

45. Shekelle PG, Motala A, Johnsen B: **AHRQ Methods for Effective Health Care**. In: *Assessment of a Method to Detect Signals for Updating Systematic Reviews.* edn. Rockville (MD): Agency for Healthcare Research and Quality (US); 2014.

46. Shekelle PG, Newberry SJ, Wu H, Suttorp M, Motala A, Lim YW, Balk EM, Chung M, Yu WW, Lee J *et al*: **AHRQ Methods for Effective Health Care**. In: *Identifying Signals for Updating Systematic Reviews: A Comparison of Two Methods.* edn. Rockville (MD): Agency for Healthcare Research and Quality (US); 2011.

47. Shekelle P, Eccles MP, Grimshaw JM, Woolf SH: **When should clinical guidelines be updated?** *Bmj* 2001, **323**(7305):155-157.

48. Shojania KG, Sampson M, Ansari MT, Ji J, Doucette S, Moher D: **How quickly do systematic reviews go out of date? A survival analysis**. *Ann Intern Med* 2007, **147**(4):224-233.

49. Shojania KG, Sampson M, Ansari MT, Ji J, Garritty C, Rader T, Moher D: **AHRQ Technical Reviews**. In: *Updating Systematic Reviews.* edn. Rockville (MD): Agency for Healthcare Research and Quality (US); 2007.

50. Soll RF: **Updating reviews: the experience of the Cochrane Neonatal Review Group**. *Paediatr Perinat Epidemiol* 2008, **22 Suppl 1**:29-32.

51. Sutton AJ, Donegan S, Takwoingi Y, Garner P, Gamble C, Donald A: **An encouraging assessment of methods to inform priorities for updating systematic reviews**. *J Clin Epidemiol* 2009, **62**(3):241-251.

52. Takwoingi Y, Hopewell S, Tovey D, Sutton AJ: **A multicomponent decision tool for prioritising the updating of systematic reviews**. *Bmj* 2013, **347**:f7191.

53. U.S. Preventive Services Task Force: **US Preventive Services Task Force Procedure Manual**. In*.*, edn.; 2015.

54. Welsh E, Stovold E, Karner C, Cates C: **Cochrane Airways Group reviews were prioritized for updating using a pragmatic approach**. *J Clin Epidemiol* 2015, **68**(3):341-346.

55. Working Group for CPG Updates: **Updating Clinical Practice Guidelines in the National Health System: Methodology Handbook**. In: *Clinical Practice Guidelines in the National Health System: I+CS No2007/02-01.* edn.: National Health System Quality Plan of the Spanish Ministry of Health and Social Policy, Aragon Health Sciences Institute (I+CS); 2009.

56. World Health Organization: **WHO handbook for guideline development - 2nd edition**. In*.*, edn. Geneva, Switzerland: WHO Press; 2014.

57. Akl EA, Meerpohl JJ, Elliott J, Kahale LA, Schünemann HJ: **Living systematic reviews: 4. Living guideline recommendations**. *J Clin Epidemiol* 2017, **91**:47-53.

58. El Mikati IK, Khabsa J, Harb T, Khamis M, Agarwal A, Pardo-Hernandez H, Farran S, Khamis AM, El Zein O, El-Khoury R *et al*: **A Framework for the Development of Living Practice Guidelines in Health Care**. *Ann Intern Med* 2022, **175**(8):1154-1160.

59. Bragge P, Allison DJ, Mehta S, Guy S, Loh E: **A pilot living clinical practice guidelines approach was feasible and acceptable to guideline panel members**. *J Clin Epidemiol* 2022, **143**:22-29.
